# Supplementary material for: Deprivation-Induced Homeostatic Spine Scaling In Vivo Is Localized to Dendritic Branches that Have Undergone Recent Spine Loss
Source: Neuron. 2017 Nov 15;96(4):871–882.e5. doi: 10.1016/j.neuron.2017.09.052 (PMC5697914; doi:10.1016/j.neuron.2017.09.052)
Supplement: Document S1. Figures S1–S3 and Tables S1–S3 [file mmc1.pdf]

Neuron, Volume *96*

Supplemental Information

Deprivation-Induced Homeostatic Spine Scaling *In Vivo* Is Localized to Dendritic Branches that Have Undergone Recent Spine Loss

Samuel J. Barnes, Eleonora Franzoni, R. Irene Jacobsen, Ferenc Erdelyi, Gabor Szabo, Claudia Clopath, Georg B. Keller, and Tara Keck

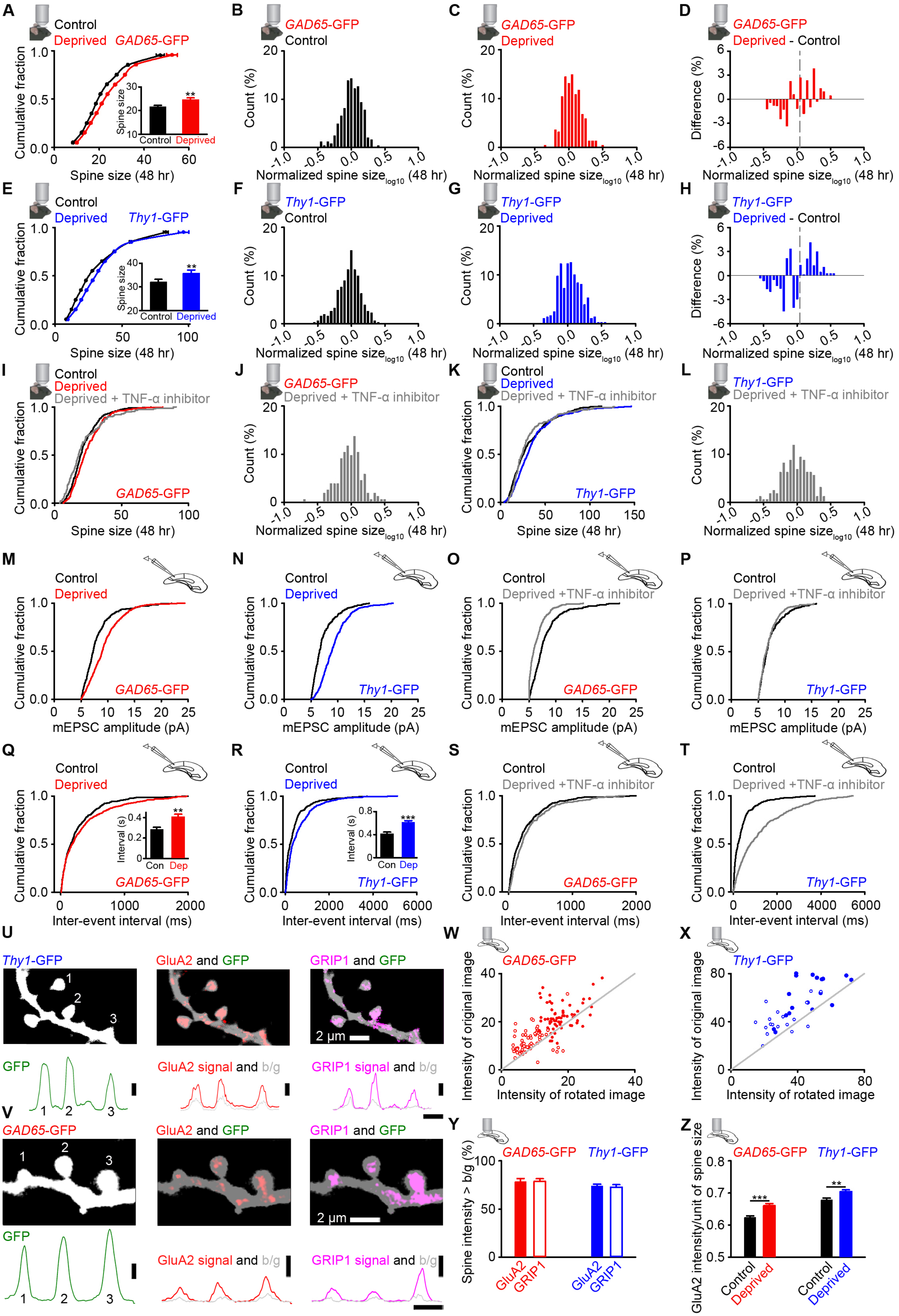

Supplementary Figure 1

Figure S1, Related to Figure 1.

A,E,I,K) Cumulative distribution of raw spine size values for inhibitory (A,I) and excitatory (E,K) neurons *in vivo* 48 hours after deprivation in control mice (black), deprived mice (red/blue) and deprived mice injected with the TNF-α inhibitor (I,K,gray). (Statistics on distributions of all spines. I, Control versus deprived, $p=0.011$; Deprived versus deprived+TNF-α inhibitor, $p<0.001$; Control versus deprived+TNF-α inhibitor, $p=0.665$; One-Way ANOVA on Ranks with post-hoc test. K, Control versus deprived, $p=0.021$; Deprived versus deprived+TNF-α inhibitor, $p=0.013$; Control versus deprived+TNF-α inhibitor, $p=0.998$; One-Way ANOVA on Ranks with post-hoc test). Insets in A and E show average spine size (Control versus deprived: A, $p=0.002$; E, $p=0.006$, t-test on log-transformed data).

B,C,F,G,J,L) Distribution of normalized (48 hours normalized to baseline) spine size values following a log-transform for inhibitory (B,C,J) and excitatory (F,G,L) neurons *in vivo* 48 hours after (sham) enucleation in control mice (B,F, black), deprived mice (C,G, red/blue) and deprived mice injected with the TNF-α inhibitor (J,L, gray). (Statistics on distribution of all spines. B,C,J, Control versus deprived, $p=0.008$; Control versus Deprived+TNF-α inhibitor, $p=0.064$; Two-Way ANOVA with post-hoc test on log-transformed data. F,G,L, Control versus deprived, $p<0.001$; Control versus deprived+TNF-α inhibitor, $p=0.923$; Two-Way ANOVA with post-hoc test on log-transformed data – Note that these are the same statistics as in Table S1 for Fig. 1D,E with the Two-Way ANOVA).

D,H) Distribution of the difference in control and deprived (48 hours) log-transformed spine size distributions between for inhibitory (D) and excitatory (H) neurons. Gray dashed line shows 1.1 threshold for spine size increase.

M,N,O,P) Cumulative distribution of mEPSC amplitude for inhibitory (M,O) and excitatory (N,P) neurons in slices prepared 48 hours after (sham) deprivation from control mice (M-P black), deprived mice (M-N, red/blue) and deprived mice injected with the TNF-α inhibitor (O-P, gray). (Statistics on distribution of all events. M,O, Control versus deprived, $p<0.001$; Control versus deprived+TNF-α inhibitor, $p<0.001$, One-Way ANOVA on Ranks with post-hoc test. N,P, Control versus deprived, $p<0.001$; Control versus deprived+TNF-α inhibitor, $p=0.456$, One-Way ANOVA on Ranks with post-hoc test). For panels M,O, control $n=9$ cells, deprived $n=16$ cells,

deprived+TNF-α inhibitor n=10 cells. For panels N,P, control n=10 cells, deprived n=15 cells, deprived+TNF-α inhibitor n=13 cells.

Q,R,S,T) Distribution of mEPSC inter-event intervals for inhibitory (Q,S) and excitatory (R,T) neurons in slices prepared 48 hours after (sham) deprivation from control mice (Q-T black), deprived mice (Q-R, red/blue) and deprived mice injected with the TNF-α inhibitor (S-T, gray). (Statistics on distributions of all events. Q,S, Control versus deprived, p=0.007; Control versus deprived+TNF-α inhibitor, p=0.044, One-Way ANOVA on Ranks with post-hoc test. R,T, Control versus deprived, p<0.001; Control versus deprived+TNF-α inhibitor, p<0.001, One-Way ANOVA on Ranks with post-hoc test). Insets, inter-event interval average (Control versus deprived: Q, p=0.007; R, p<0.001, t-test on log-transformed data). For panels Q,S, control n=9 cells, deprived n=16 cells, deprived+TNF-α inhibitor n=10 cells. For panels R,T, control n=10 cells, deprived n=15 cells, deprived+TNF-α inhibitor n=13 cells.

U,V) Top, example images of a dendritic section from either an excitatory (U) or inhibitory (V) neuron in slices prepared from mice 48 hours after deprivation. Example images show immunohistochemistry against GFP (left), GluA2 (middle) and GRIP1 (right). Scale bar: 2 μm. Bottom, fluorescence intensity traces measured in the numbered spines in each example of GFP (green), GluA2 (red), GRIP1 (magenta) and background (gray; 90 degree rotation of fluorescence image, not shown). Scale bars: 2 μm (horizontal) and 25 intensity units (vertical).

W,X) Spine intensity values of GluA2 (filled) and GRIP1 (open) for a subset of dendritic spines in inhibitory (W) and excitatory (X) neurons measured for the same spine in either the original immunohistochemical images or images rotated by 90 degrees. Each circle is the measure from an individual spine. (GluA2: Original versus rotated, W, p<0.001; X, p<0.001. GRIP1: Original versus rotated, W, p<0.001; X, p<0.001, Wilcoxon signed rank test).

Y) Percentage of imaged spines showing GluA2 (filled) and GRIP1 (open) intensity values that are greater than background (calculated from the 90 degree rotated immunohistochemistry image) for inhibitory (red) or excitatory (blue) cells in slices prepared from animals 48 hours after enucleation. Inhibitory, n=79 branches. Excitatory, n=62 branches.

Z) Spine GluA2 intensity normalized to background, then normalized to individual spine size for inhibitory (red) and excitatory (blue) neurons in slices prepared 48 hours after deprivation (red/blue) and from control mice (black). (Control versus deprived: Inhibitory, $p<0.001$; Excitatory, $p=0.001$, Mann Whitney Rank Sum Test). Inhibitory: deprived, n=998 spines; control, n=1887 spines. Excitatory: deprived, n=1025 spines; control, n=1450 spines.

Insets for summary data panels: mouse with objective is *in vivo* imaging experiment, slice with objective is *in vitro* imaging experiment, slice with electrode is *in vitro* electrophysiology experiment. For all panels, **$p<0.01$; ***$p<0.001$. Error bars, mean and s.e.m. For clarity, crossing axons have been removed from all images.

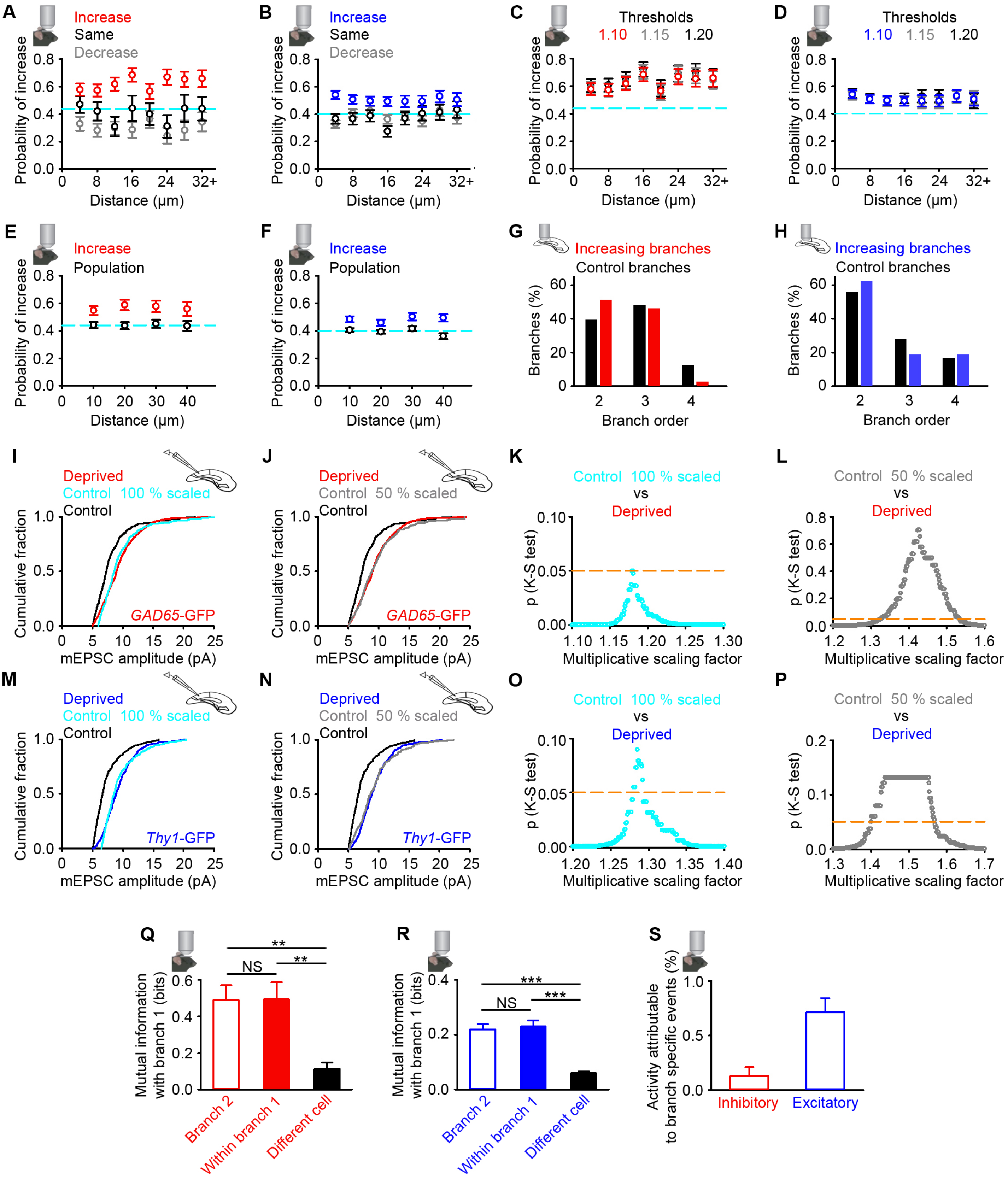

Supplementary Figure 2

Figure S2, Related to Figure 2.

A,B) Same cluster analysis as in Fig. 2B-C, but grouped by the behavior of the spine Sp_0 which either increases (blue/red, $Sp_0>1.1$), stays a similar size ('same', black, $1.1>Sp_0>0.9$) or decreases in size (gray, $Sp_0<0.9$). For each Sp_0 behavior, the fraction of spines at different distances from spine Sp_0 (4 µm bins) increasing for inhibitory (A, Interaction between behaviour and position, p=0.829; Behavior: Increase versus same, p<0.001; Increase versus decrease, p<0.001, Two-Way ANOVA with post-hoc test) and excitatory neurons (B, Interaction between behaviour and position, p=0.726; Behavior: Increase versus same, p<0.001; Increase versus decrease, p<0.001, Two-Way ANOVA with post-hoc test). Data are shown for 48 hours after enucleation normalized to baseline value for each spine. Cyan dashed line depicts proportion of all spines increasing. For panel A, n=31 branches. For panel B, n=24 branches.

C,D) For all Sp_0 spines exceeding an increased size threshold (1.1, 1.15, 1.2), the fraction of all neighbors a given distance (in 4 µm bins) away on the dendritic branch that also exceed the same size increase threshold as the Sp_0 spine (1.1, 1.15, 1.2) measured 48 hours post-enucleation and normalized to baseline for individual spines from branches in inhibitory (C, Threshold and distance, p=0.487, Two-Way ANOVA) and excitatory neurons (D, Threshold and distance, p=0.845, Two-Way ANOVA). Cyan dashed line depicts proportion of all spines increasing. For panel C, n=31 branches. For panel D, n=24 branches.

E,F) Same cluster analysis as in Fig. S2A-D and Fig. 2B-C for spines exceeding a threshold of 1.1 size increase within a given distance (in 10 µm bins) of a spine, Sp_0, 48 hours after enucleation normalized to baseline value. For Sp_0s that either exceed threshold 1.1 (red/blue) or the population average including all spines (black) for dendritic branches from inhibitory (E, Increasing versus population, p<0.001; 10 µm, p=0.001; 20 µm, p<0.001; 30 µm, p=0.011; 40 µm, p=0.036; Within increasing, 10 µm versus 40 µm, p=0.854; Two-Way ANOVA with post-hoc test) and excitatory neurons (F, Increasing versus population, p<0.001; 10 µm, p=0.002; 20 µm, p=0.013; 30 µm, p=0.004; 40 µm, p<0.001; Within increasing, 10 µm versus 40 µm, p=0.767; Two-Way ANOVA with post-hoc test). Cyan dashed line depicts proportion of all spines increasing. For panel E, n=31 branches. For panel F, n=24 branches.

G,H) Distribution of branch order (percentage of branches for each branch order out of all branches within a condition) for either inhibitory (G) or excitatory (H) neurons for branches showing an increase in spine size

(red/blue) and branches from sham enucleated control animals (black) from the same blindly collected and analyzed dataset. For panel G, control n=148 branches, deprived n=79 branches. For panel H, control n=62 branches, deprived n=62 branches.

I,J,M,N) Distribution of mEPSC amplitude for inhibitory neurons (I-J) and excitatory (M-N) neurons in slices prepared 48 hours after deprivation from control mice (black) with either 100% (I,M) or 50% (J,N) of mEPSC events from the control distribution multiplicatively scaled (cyan 100%, gray 50%) compared to the deprived distribution (red/blue). Data are shown for the best fit scaling factor (I, 1.179; J, 1.424; M, 1.285; N, 1.46). For panels I,J, control n=9 cells, deprived n=16 cells. For panels M,N, control n=10 cells, deprived n=15 cells.

K,L,O,P) Resulting p-values from Kolomogrov-Smirnov (K-S) tests (circles) for the multiplicative scaling factors used to scale either the entire (100%) control distribution (K,O) or 50% of the control distribution (L,P) and comparing it to the deprived distribution for either inhibitory (K-L) or excitatory neurons (O-P). Orange dashed line shows where p=0.05 (5% confidence interval). We tested the goodness of fit to the experimentally measured deprived distribution for the 50% scaled and for the 100% scaled control distributions using a Kullback-Leibler divergence statistic. We found a lower Kullback-Leibler value for the 50% scaled distribution, corresponding to less information lost and a better fit to the deprived distribution in both inhibitory (100% Scaled = 0.129 ± 0.003, 50% Scaled = 0.037 ± 0.001 Bits, p<0.001, t-test on Kullback-Leibler divergence scores) and excitatory (100% Scaled = 0.177 ± 0.001, 50% Scaled = 0.059 ± 0.001 Bits, p<0.001, t-test on Kullback-Leibler divergence scores) neurons.

Q,R) For GCaMP6f functional imaging measurements in behaving mice, the mutual information calculation with branch 1 for a dendritic branch sharing a branch point (Branch 2), a neighboring region 10 µm apart on the same dendritic branch (Within branch 1) or a dendrite in the same imaging region, but on a different cell (Different cell) in inhibitory neurons 24 hours post-enucleation (Q) or excitatory neurons 4 hours post-enucleation (R) (Q, red open (Branch 1-Branch 2) versus red filled (Branch 1-Within branch 1), p=0.970; red open versus black filled (Branch 1-Different cell), p=0.003; red filled versus black filled, p=0.005; R, blue open (Branch 1-Branch 2) versus blue filled (Branch 1-Within branch 1), p=0.686; blue open versus black filled (Branch 1-Different cell), p<0.001; blue filled versus black filled, p<0.001, One-Way ANOVA with post-hoc test). For panel Q, n=7 branch-1 cells, n=7 different cells. For panel R, n=6 branch-1 cells, n=6 different cells.

S) Activity attributable to branch specific events as a percentage of total overall activity in the branch for inhibitory (red) and excitatory (blue) branches in deprived animals. Inhibitory, n=7 branch pairs. Excitatory, n=6 branch pairs.

Insets for summary data panels: mouse with objective is *in vivo* imaging experiment, slice with objective is *in vitro* imaging experiment, slice with electrode is *in vitro* electrophysiology experiment. For all panels, NS=no significance; **$p<0.01$; ***$p<0.001$. Error bars, mean and s.e.m.

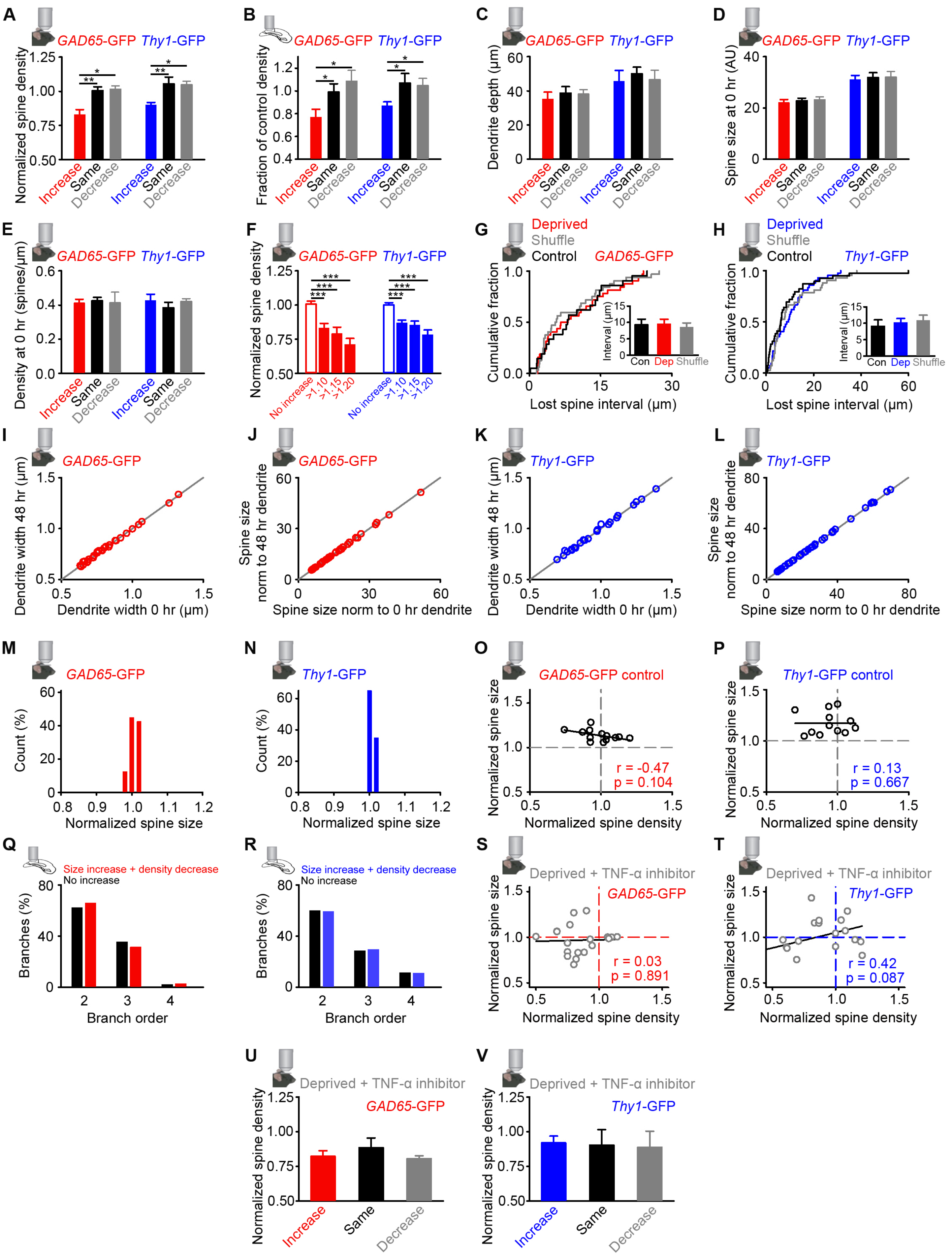

Supplementary Figure 3

Figure S3, Related to Figure 3.

A) Spine density 48 hours post-enucleation normalized to baseline for individual branches whose average post-enucleation spine size increases (red/blue, >1.1), stays a similar average size ('same', black, between 0.9 and 1.1) or decreases in size (gray, <0.9) relative to their individual baseline in inhibitory (Increase versus same, p=0.002; Increase versus decrease, p=0.021, One-Way ANOVA with post-hoc test) and excitatory neurons (Increase versus same, p=0.005; Increase versus decrease, p=0.028, One-Way ANOVA with post-hoc test). Inhibitory, n=31 branches; Excitatory, n=24 branches.

B) Fraction of control spine density (fraction of average control value) for individual branches whose spines increase in size (red/blue, >1.1), stay a similar average size ('same', black, between 0.9 and 1.1) or decrease in size (gray, <0.9) relative to control values (see Methods) measured from slices prepared from mice 48 hours after enucleation in inhibitory (Increase versus same, p=0.047; Increase versus decrease, p=0.010, One-Way ANOVA with post-hoc test) and excitatory neurons (Increase versus same, p=0.026; Increase versus decrease, p=0.049, One-Way ANOVA with post-hoc test). Inhibitory, n=79 branches; Excitatory, n=62 branches.

C) Cortical depth (distance from the surface of the brain to branch midpoint) of dendritic branches whose average post-enucleation spine size increases (red/blue, >1.1), stays a similar average size ('same', black, between 0.9 and 1.1) or decreases in size (gray, <0.9) relative to their individual baseline after enucleation in inhibitory (red, p=0.812, One-Way ANOVA) and excitatory (blue, p=0.846, One-Way ANOVA) neurons in deprived animals measured with *in vivo* imaging. Inhibitory, n=31 branches. Excitatory, n=24 branches.

D) Average stable spine size prior to deprivation for dendritic branches whose average post-enucleation spine size increases (red/blue, >1.1), stays a similar average size ('same', black, between 0.9 and 1.1) or decreases in size (gray, <0.9) relative to their individual baseline in inhibitory (red, p=0.427, One-Way ANOVA on ranks) and excitatory (blue, p=0.373, One-Way ANOVA on ranks) neurons measured with *in vivo* imaging. Note that this measure is for stable spines that will not be lost following deprivation. Inhibitory, n=31 branches. Excitatory, n=24 branches.

E) Spine density prior to deprivation for dendritic branches whose average post-enucleation spine size increases (red/blue, >1.1), stays a similar average size ('same', black, between 0.9 and 1.1) or decreases in size (gray, <0.9)

relative to their individual baseline in inhibitory (red, p=0.927, One-Way ANOVA) and excitatory (blue, p=0.659, One-Way ANOVA) neurons measured with *in vivo* imaging. Inhibitory, n=31 branches. Excitatory, n=24 branches.

F) Spine density at 48 hours post-enucleation normalized to baseline within dendrite for branches that do not show average spine size increases (<1.1, individual spines 48 hours post-enucleation normalized to baseline, then averaged across the dendritic branch) and those that show an average spine size change of greater than 1.1, 1.15 or 1.2 for inhibitory (red) and excitatory (blue) neurons. (Inhibitory: No increase versus >1.1, p<0.001; No increase versus >1.15, p<0.001; No increase versus >1.2, p<0.001. Excitatory: No increase versus >1.1, p<0.001; No increase versus >1.15, p<0.001; No increase versus >1.2, p<0.001, One-Way ANOVA with post-hoc test). Inhibitory, n=31 branches. Excitatory, n=52 branches (includes data from Fig. 1E and Fig. 3E).

G,H) Cumulative distribution of interspine intervals for lost spines across all time points in inhibitory (G) and excitatory (H) neurons in deprived (red/blue) and control (black) animals and for deprived branches that underwent a spatial shuffle (gray). Insets, mean spine loss interspine interval. (Inhibitory: p=0.815; Excitatory: p=0.314, One-Way ANOVA on Ranks). For panel G, n=31 branches. For panel H, n=24 branches.

I,K,) Dendrite width measured prior to (0 hours) and 48 hours after enucleation for inhibitory (I) and excitatory (K) neurons. Each circle is a single dendrite. (0 hours versus 48 hours: I, p=0.197; K, p=0.984, paired t-test). For panel I, n=31 branches. For panel K, n=24 branches.

J,L) Spine size measured at 0 hours normalized to the adjacent dendrite taken from the image at either 0 hours or 48 hours after enucleation. Each circle is a single spine. (0 hours versus 48 hours: J, p=0.477; L, p=0.421, paired t-test). For panel J, n=40 spines. For panel L, n=40 spines.

M,N) Distribution of 0 hour spine size measured using the dendrite at 48 hours normalized to the same 0 hour spine size measured using the dendrite at 0 hours in inhibitory (M, 1.000 ± 0.002 normalized spine size) and excitatory neurons (N, 1.000 ± 0.001 normalized spine size). For panel M, n=40 spines. For panel N, n=40 spines.

O,P) Spine density versus average spine size normalized to baseline for dendritic branches in inhibitory (O) and excitatory (P) neurons that undergo an average increase in spine size (but not necessarily "increasing branches", see Methods) in sham-enucleated animals measured with chronic *in vivo* imaging. Spine density and size are measured 48 hours (O) or 8 hours (P) after sham-enucleation and normalized to baseline for individual branches (density) and individual spines (size). Normalized spine size is then averaged across the branch. (O, $r=-0.47$, $p=0.104$; P, $r=0.13$, $p=0.667$, Pearson's correlation). For panel O, n=13 branches. For panel P, n=12 branches.

Q,R) Distribution of branch order (percentage of branches for each branch order out of all branches within a condition) for either inhibitory (Q) or excitatory (R) neurons measured from slices prepared from mice 48 hours after enucleation. Branches showing an increase in spine size and a decrease in spine density (red/blue) and branches that do not show an increase (black) from the same blindly collected and analyzed dataset. For panel Q, n=79 branches. For panel R, n=62 branches.

S,T) For deprived animals that also received injections of the TNF-α inhibitor, normalized spine density versus average normalized stable spine size measured using repeated *in vivo* imaging. Measured 48 hours after enucleation and normalized to baseline for individual dendritic branches (density) or individual spines (size). Normalized spine size was then averaged for each branch. Dendrites in inhibitory (S) and excitatory (T) neurons (S, $r=0.03$, $p=0.891$; T, $r=0.42$, $p=0.087$, Pearson's correlation). For panel S, n=19 branches. For panel T, n=18 branches.

U,V) Spine density in deprived animals that were injected with the TNF-α inhibitor 48 hours post-enucleation normalized to baseline for individual branches whose average spine size increases (red/blue, >1.1), stays a similar average size ('same', black, between 0.9 and 1.1) or decreases in size (gray, <0.9) in inhibitory (U, Increase versus same, $p=0.658$; Increase versus decrease, $p=0.676$, One-Way ANOVA with post-hoc test) and excitatory (V, Increase versus same, $p=0.895$; Increase versus decrease, 0.809, One-Way ANOVA with post-hoc test) neurons. For panel U, n=19 branches. For panel V, n=18 branches.

Insets for summary data panels: mouse with objective is *in vivo* imaging experiment, slice with objective is *in vitro* imaging experiment. For all panels, *$p<0.05$; **$p<0.01$; ***$p<0.001$. Error bars, mean and s.e.m.

Statistical Comparisons for Figure 1			Inhibitory		Excitatory	
**Panel**	**Comparison**	**Test**	**p value**	**n value**	**p value**	**n value**
**1D,E**	Time and experimental condition, overall interaction	Two-Way ANOVA with post-hoc test on log-transformed data	p = 0.015	control n=40 branches<br>deprived n=31 branches<br>deprived+TNF-α inhibitor n=19 branches	p = 0.006	control n=34 branches<br>deprived n=24 branches<br>deprived+TNF-α inhibitor n=18 branches
	Control vs deprived at 0 hrs		p = 0.359		p = 0.417	
	Control vs deprived + TNF-α inhibitor at 0 hrs		p = 0.312		p = 0.588	
	Deprived vs deprived + TNF-α inhibitor at 0 hrs		p = 0.670		p = 0.291	
	Control vs deprived at 24 hrs		p = 0.560		p < 0.001	
	Control vs deprived + TNF-α inhibitor at 24 hrs		p = 0.708		p = 0.627	
	Deprived vs deprived + TNF-α inhibitor at 24 hrs		p = 0.512		p = 0.008	
	Control vs deprived at 48 hrs		p = 0.008		p < 0.001	
	Control vs deprived + TNF-α inhibitor at 48 hrs		p = 0.064		p = 0.923	
	Deprived vs deprived + TNF-α inhibitor at 48 hrs		p < 0.001		p < 0.001	
**1D,E**	0 vs 24 hrs deprived	One-Way Repeated Measures ANOVA with post-hoc test on log-transformed data	p = 0.638	control n=40 branches<br>deprived n=31 branches<br>deprived+TNF-α inhibitor n=19 branches	p = 0.045	control n=34 branches<br>deprived n=24 branches<br>deprived+TNF-α inhibitor n=18 branches
	0 vs 24 hrs deprived+TNF-α inhibitor		p = 0.655		p = 0.959	
	0 vs 24 hrs control		p = 0.224		p = 0.264	
	0 vs 48 hrs deprived		p = 0.021		p = 0.003	
	0 vs 48 hrs deprived+TNF-α inhibitor		p = 0.204		p = 0.624	
	0 vs 48 hrs control		p = 0.092		p = 0.061	
**1G**	Control vs deprived	One-Way ANOVA with post-hoc test	p = 0.015	control n=9 cells<br>deprived n=16 cells<br>deprived+TNF-α inhibitor n=10 cells		
	Deprived vs deprived + TNF-α inhibitor		p < 0.001			
	Control vs deprived + TNF-α inhibitor		p = 0.237			
**1H**	Control vs deprived	One-Way ANOVA on Ranks with post-hoc test			p < 0.001	control n=10 cells<br>deprived n=15 cells<br>deprived+TNF-α inhibitor n=13 cells
	Deprived vs deprived + TNF-α inhibitor				p = 0.031	
	Control vs deprived + TNF-α inhibitor				p = 0.325	
**1J**	Control vs deprived	t-test	p = 0.029	control n=148 branches<br>deprived n=79 branches	p = 0.031	control n=62 branches<br>deprived n=62 branches
**1K**	Control vs deprived	t-test	p = 0.021	control n=148 branches<br>deprived n=79 branches	p = 0.043	control n=62 branches<br>deprived n=62 branches

Table S1. Statistical comparisons for Figure 1, Related to Figure 1.

Statistical Comparisons for Figure 2			Inhibitory		Excitatory	
Panel	Comparison	Test	p value	n value	p value	n value
2B,C	Increasing vs population over all distance	Two-Way ANOVA with post-hoc test	$p < 0.001$	n = 31 branches	$p < 0.001$	n = 24 branches
	Increasing vs population at 4 μm		$p = 0.039$		$p < 0.001$	
	Increasing vs population at 8 μm		$p = 0.018$		$p = 0.010$	
	Increasing vs population at 12 μm		$p = 0.002$		$p = 0.012$	
	Increasing vs population at 16 μm		$p = 0.002$		$p = 0.002$	
	Increasing vs population at 20 μm		$p = 0.035$		$p = 0.005$	
	Increasing vs population at 24 μm		$p < 0.001$		$p = 0.008$	
	Increasing vs population at 28 μm		$p = 0.049$		$p = 0.026$	
	Increasing vs population at 32+ μm		$p = 0.012$		$p = 0.029$	
	Increasing vs population at different branch		$p = 0.558$		$p = 0.438$	
	Within increasing: 4 μm vs 28 μm		$p = 0.996$		$p = 0.998$	
	Increasing vs shuffle at 4 μm		$p = 0.875$		$p = 0.141$	
	Increasing vs shuffle at 8 μm		$p = 0.635$		$p = 0.709$	
	Increasing vs shuffle at 12 μm		$p = 0.552$		$p = 0.994$	
	Increasing vs shuffle at 16 μm		$p = 0.207$		$p = 0.682$	
	Increasing vs shuffle at 20 μm		$p = 0.666$		$p = 0.887$	
	Increasing vs shuffle at 24 μm		$p = 0.996$		$p = 0.509$	
	Increasing vs shuffle at 28 μm		$p = 0.635$		$p = 0.397$	
	Increasing vs shuffle at 32+ μm		$p = 0.688$		$P = 0.414$	
2D	One vs both	One-Way ANOVA with post-hoc test	$p < 0.001$	n = 94 branch pairs	$p = 0.019$	n = 138 branch pairs
	One vs neither		$p = 0.002$		$p = 0.003$	
2E	One vs both	One-Way ANOVA with post-hoc test	$p = 0.029$	n = 100 branch pairs	$p = 0.031$	n = 72 branch pairs
	One vs neither		$p = 0.027$		$p = 0.022$	
2H,I	Branch 1 and branch 2	Spearman's correlation	$r = 0.90$ $p < 0.001$	example	$r = 0.91$ $p < 0.001$	example
	Branch 1 and different cell		$r = -0.16$ $p < 0.001$		$r = 0.19$ $p < 0.001$	
2J,K	Branch 2 vs within branch 1 vs different cell	One-Way repeated measures ANOVA	$p = 0.264$	n = 7 branch 1 cells n = 7 different cells	$p = 0.145$	n = 6 branch 1 cells n = 6 different cells
2L,M	Branch 2 vs within branch 1	One-Way ANOVA with post-hoc test	$p = 0.746$	n = 7 branch 1 cells n = 7 different cells	$p = 0.900$	n = 6 branch 1 cells n = 6 different cells
	Within branch 1 vs different cell		$p < 0.001$		$p < 0.001$	
	Branch 2 vs different cell		$p < 0.001$		$p < 0.001$	
2O	Branch specific vs all events	t-test	$p < 0.001$	n = 7 branch pairs n = 166 total calcium events	$p < 0.001$	n = 6 branch pairs n=140 total calcium events

**2P**	Branch specific vs all events	t-test	$p < 0.001$	n=7 branch pairs n=166 total calcium events	$p < 0.001$	n=6 branch pairs n=140 total calcium events
**2Q**	Deprived vs control	t-test	$p = 0.982$	deprived n = 7 branch pairs control n = 7 branch pairs	$p = 0.945$	deprived n = 6 branch pairs control n = 7 branch pairs

Table S2. Statistical comparisons for Figure 2, Related to Figure 2. For panel 2D, there is a higher n for branch pair comparisons because some cells have more than two branches and for excitatory cells, we have included data from Fig. 1E and Fig. 3E.

Statistical Comparisons for Figure 3			Inhibitory		Excitatory	
**Panel**	**Comparison**	**Test**	**p value**	**n value**	**p value**	**n value**
**3C**	Increase vs no increase	t-test	p < 0.001	n = 31 branches	p = 0.004	n = 24 branches
**3D**	Density 24 hrs	Repeated measures ANOVA with post-hoc test	p < 0.001	n = 31 branches		
	Density 48 hrs		p < 0.001			
	Size 24 hrs	Repeated measures ANOVA with post-hoc test on log-transformed data	p = 0.638			
	Size 48 hrs		p = 0.021			
**3E**	Density 4 hrs	Repeated measures ANOVA with post-hoc test			p < 0.001	n = 28 branches
	Density 8 hrs				p < 0.001	
	Size 4 hrs	Repeated measures ANOVA with post-hoc test on log-transformed data			p = 0.570	
	Size 8 hrs				p = 0.002	
**3F,G**	Normalized spine size vs normalized spine density	Pearson's correlation	r = -0.65<br>p < 0.001	n = 31 branches	r = -0.65<br>p < 0.001	n = 28 branches
**3I**	Increase vs no increase	t-test	p = 0.023	n = 79 branches	p = 0.043	n = 62 branches
**3J,K**	Fraction of control size vs fraction of control density	Pearson's correlation	r = -0.33<br>p < 0.001	n = 79 branches	r = -0.30<br>p = 0.032	n = 62 branches
**3L,M**	Density 24 hrs	Repeated measures ANOVA with post-hoc test	p = 0.003	n = 19 branches	p = 0.359	n = 18 branches
	Density 48 hrs		p < 0.001		p = 0.027	
**3N**	Increase vs no increase	t-test	p = 0.771	n = 19 branches	p = 0.832	n = 18 branches

Table S3. Statistical comparisons for Figure 3, Related to Figure 3.
