# Supplementary material for: Deprivation-Induced Homeostatic Spine Scaling In Vivo Is Localized to Dendritic Branches that Have Undergone Recent Spine Loss
Source: Neuron. 2017 Nov 15;96(4):871–882.e5. doi: 10.1016/j.neuron.2017.09.052 (PMC5697914; doi:10.1016/j.neuron.2017.09.052)
Supplement: Document S2. Article plus Supplemental Information [file mmc2.pdf]

Neuron

Article

Deprivation-Induced Homeostatic Spine Scaling *In Vivo* Is Localized to Dendritic Branches that Have Undergone Recent Spine Loss

Highlights

- Inhibitory and excitatory neurons exhibit TNF-α-dependent spine size increases
- Spine size increases occur in a subset of dendritic branches after deprivation
- Increases in spine size are correlated with local spine loss within a branch
- Simulations show that branch-specific plasticity increases information processing

Authors

Samuel J. Barnes, Eleonora Franzoni, R. Irene Jacobsen, ..., Claudia Clopath, Georg B. Keller, Tara Keck

Correspondence

t.keck@ucl.ac.uk

In Brief

Barnes et al. show that TNF-α-dependent spine size increases are correlated with recent spine loss within dendritic branches in both inhibitory and excitatory cortical neurons *in vivo* following visual deprivation. Branch-specific plasticity increases information processing relative to global plasticity.

Barnes et al., 2017, Neuron *96*, 871–882
November 15, 2017 © 2017 The Author(s). Published by Elsevier Inc.
https://doi.org/10.1016/j.neuron.2017.09.052

CellPress

CellPress

Deprivation-Induced Homeostatic Spine Scaling *In Vivo* Is Localized to Dendritic Branches that Have Undergone Recent Spine Loss

Samuel J. Barnes,[1,2,6] Eleonora Franzoni,[1] R. Irene Jacobsen,[1,2,7] Ferenc Erdelyi,[3] Gabor Szabo,[3] Claudia Clopath,[4] Georg B. Keller,[5] and Tara Keck[1,2,8,*]

[1]Department of Neuroscience, Physiology, and Pharmacology, University College London, 21 University Street, London WC1E 6DE, UK
[2]MRC Centre for Developmental Neurobiology, King's College London, New Hunt's House 4th Floor, London SE1 1UL, UK
[3]Medical Gene Technology Unit, Institute of Experimental Medicine, Hungarian Academy of Sciences, 1450 Budapest, Hungary
[4]Department of Bioengineering, Imperial College London, Bessemer Building 4th Floor, London SW7 2AZ, UK
[5]Friedrich Miescher Institute for Biomedical Research, Maulbeerstrasse 66, Basel 4058, Switzerland
[6]Present address: Division of Brain Sciences, Department of Medicine, Imperial College London, Hammersmith Hospital Campus, Du Cane Road, London W12 0NN, UK
[7]Present Address: Kavli Institute for Systems Neuroscience and Centre for Neural Computation, Norwegian University of Science and Technology, Olav Kyrres Gate 9, 7030 Trondheim, Norway
[8]Lead Contact
*Correspondence: t.keck@ucl.ac.uk

https://doi.org/10.1016/j.neuron.2017.09.052

SUMMARY

Synaptic scaling is a key homeostatic plasticity mechanism and is thought to be involved in the regulation of cortical activity levels. Here we investigated the spatial scale of homeostatic changes in spine size following sensory deprivation in a subset of inhibitory (layer 2/3 *GAD65*-positive) and excitatory (layer 5 *Thy1*-positive) neurons in mouse visual cortex. Using repeated *in vivo* two-photon imaging, we find that increases in spine size are tumor necrosis factor alpha (TNF-α) dependent and thus are likely associated with synaptic scaling. Rather than occurring at all spines, the observed increases in spine size are spatially localized to a subset of dendritic branches and are correlated with the degree of recent local spine loss within that branch. Using simulations, we show that such a compartmentalized form of synaptic scaling has computational benefits over cell-wide scaling for information processing within the cell.

INTRODUCTION

Following a reduction in activity resulting from sensory deprivation, excitatory synapses have been shown to strengthen, which is thought to facilitate the restoration of activity levels (Hengen et al., 2013, 2016; Keck et al., 2013; Wallace and Bear, 2004). There are two well-studied mechanisms that lead to strengthening of synapses: homeostatic mechanisms, such as synaptic scaling (Turrigiano et al., 1998), and Hebbian mechanisms, such as long-term potentiation (LTP). Hebbian processes occur over small spatial scales, on the order of single synapses or small groups of neighboring synapses (Harvey et al., 2008), and are thought to strengthen spared inputs following sensory deprivation and facilitate cortical reorganization (Cheetham et al., 2012; Feldman, 2000). In contrast, synaptic scaling is believed to occur across a larger, cell-wide spatial scale (Turrigiano et al., 1998) and is potentially implemented through global changes in α-amino-3-hydroxy-5-methyl-4-isoxazolepropionic acid receptor (AMPAR) properties (Makino and Malinow, 2011; Turrigiano et al., 1998). However, studies in reduced preparations have demonstrated that synaptic scaling can be locally induced in dendritic branches (Sutton et al., 2006) and that AMPARs can be synthesized locally within branches (Ju et al., 2004). These studies suggest that scaling could be implemented independently within dendritic branches (Yu and Goda, 2009), consistent with the idea that the dendritic branch is a fundamental computational unit for the processing of neural information (Branco and Häusser, 2010; Poirazi et al., 2003).

Homeostatic mechanisms are thought to restore activity after deprivation while maintaining input-output properties of the cell. Whether these processes occur homogenously across a cell or are targeted to individual compartments *in vivo* is still unclear. This issue is of particular interest given recent evidence that inputs with similar properties cluster on dendritic branches (Iacaruso et al., 2017; Takahashi et al., 2012; Wilson et al., 2016). Therefore, input levels across the dendritic arbor may become disparate following sensory deprivation, as deprivation-induced reductions of input may vary considerably across branches. A global homeostatic strategy that modifies synaptic strengths cell-wide would perturb inputs onto branches that may have been unaffected by sensory deprivation. Thus, global scaling may come at the cost of interfering with initially unaffected input-output relationships. Understanding the spatial scales at which synapses are altered is necessary to understand how the neuron can regulate activity levels and still maintain the specific circuitry associated with memory storage established through experience.

This is an open access article under the CC BY license (http://creativecommons.org/licenses/by/4.0/).

Synaptic scaling of excitatory synapses occurs in both inhibitory and excitatory neurons following reduction of activity in reduced preparations (Hartman et al., 2006; Turrigiano et al., 1998). Synaptic scaling in inhibitory neurons will likely affect activity levels in those cells, which could in turn alter the balance between excitation and inhibition onto excitatory cells. Thus, synaptic scaling in inhibitory neurons, which has received relatively little attention to date, could have implications for overall cortical activity levels. In mouse visual cortex, we have previously described a population of inhibitory neurons that exhibit visual deprivation-induced increases in spine dynamics *in vivo* (Keck et al., 2011), suggesting that changes to spines do occur in this cell type. Our more recent work, however, shows that inhibitory neurons have a wide range of activity profiles following sensory deprivation, from cells that become functionally silent to those that increase their activity (Barnes et al., 2015). These results indicate that homeostatic recovery of activity in inhibitory neurons may be different from that in excitatory cells. Thus, whether excitatory synapses in inhibitory neurons undergo similar processes of strengthening to those seen in excitatory neurons *in vivo* following deprivation is still unclear.

Here we examined the spatial scale of spine size increases within individual cells following visual deprivation in mouse monocular visual cortex. Using *in vivo* imaging, we found that, following monocular enucleation, dendritic spines show an increase in size on both a subset of layer 2/3 inhibitory and layer 5 excitatory pyramidal neurons. When we examined the spatial extent of these spine size increases, we found that they do not occur across all dendritic branches. Instead, in both inhibitory and excitatory neurons, spine size increases were most prominent on branches that had undergone spine loss after deprivation, such that branches with greater spine loss had larger subsequent increases in the sizes of the remaining spines. Using simulations, we show that this local regulation results in higher information capacity for the neuron than global changes.

RESULTS

To examine the spatial scale of spine size changes following sensory deprivation via monocular enucleation in inhibitory and excitatory neurons, we used repeated (Figures 1A–1C, every 24 hr for 4 days) two-photon imaging of dendritic spines in the monocular visual cortex of anaesthetized adult mice expressing green fluorescent protein (GFP) either in a subset of spiny layer 2/3 inhibitory neurons (under the *GAD65* promoter; *GAD65*-GFP) (López-Bendito et al., 2004) or in layer 5 excitatory pyramidal cells (under the *Thy1* promoter; *Thy1*-GFP) (Feng et al., 2000). It is important to note that the cell bodies of these distinct cell types are in different cortical layers, but they both have dendrites in the upper layers of the cortex. Thus, for both cell types we repeatedly imaged the same dendritic spines in cortical layers 1 and 2/3 before and after deprivation and measured spine sizes as a proxy for synaptic strength, as spine size correlates with both AMPAR expression and the strength of functional synaptic responses (Noguchi et al., 2011). Here, we focus on layer 5 excitatory cells, as we have previously reported that they exhibit spine size increases following sensory deprivation (Keck et al., 2013), whereas in layer 2/3 excitatory cells, we have previously found no evidence for either net increases in spine size or synaptic scaling in adult animals (Barnes et al., 2015). We first determined whether spine size increased in layer 2/3 inhibitory neurons following monocular enucleation. We focused on a subset of inhibitory neurons that have dendritic spines, a majority of which contain excitatory synapses (Keck et al., 2011). These cells are largely (~90%) neuropeptide-Y (NPY) positive (Keck et al., 2011) and located in layer 2/3, which also technically facilitates imaging dendritic spines. In these inhibitory neurons, we found an average increase in the population spine size 48 hr after deprivation (Figures 1A, 1D, and S1A–S1D). This time course was slower than for layer 5 excitatory neurons (Figures 1B, 1E, and S1E–S1H), where the population spine size increased within 24 hr, consistent with previous results (Keck et al., 2013).

An increase in spine size in both inhibitory and excitatory neurons in response to a decrease in overall cortical activity levels could be due to either synaptic scaling or Hebbian-like processes. Before examining the spatial scale of the spine changes we observed *in vivo*, we wanted to establish whether these changes in spine size shared mechanisms with classical synaptic scaling studied in *in vitro* preparations or Hebbian LTP-like processes. Previous work has demonstrated that synaptic scaling, but not Hebbian plasticity, is dependent on TNF-α, a cell signaling cytokine released from glial cells (Kaneko et al., 2008; Stellwagen and Malenka, 2006). TNF-α is thought to be released in response to a reduction in glutamate and to support the persistent expression of synaptic scaling by upregulating AMPA receptor insertion (Steinmetz and Turrigiano, 2010; Stellwagen and Malenka, 2006). We used a pharmacological approach to inhibit TNF-α by injecting mice before and after they underwent deprivation with a dominant-negative TNF (XPRo1595, see STAR Methods; Lewitus et al., 2014, 2016) to inhibit soluble TNF-α *in vivo* (Figure 1C). Although we still observed a range of spine size changes similar to what we measured in control animals (Figures S1I–S1L), we found that the net *in vivo* spine size increases were blocked in both inhibitory (Figures 1D, S1I, and S1J) and excitatory (Figures 1E, S1K, and S1L) neurons in deprived animals that were injected with the TNF-α inhibitor, suggesting that the net increases in spine size reflect a synaptic scaling-like process.

To confirm that TNF-α inhibition also prevented functional measures of synaptic scaling, we made electrophysiological recordings of miniature excitatory postsynaptic currents (mEPSCs) in acute slices prepared from mice 48 hr after enucleation or sham-enucleation (Figure 1F). In agreement with the observed *in vivo* spine changes, we observed a multiplicative increase in mEPSC amplitude in enucleated mice in both inhibitory (Figures 1G, S1M, S2I, and S2K) and excitatory (Figures 1H, S1N, S2M, and S2O) neurons. Synaptic scaling was abolished in deprived animals when they were injected with the TNF-α inhibitor (Figures 1G and 1H, gray; Figures S1O and S1P), consistent with past work (Kaneko et al., 2008; Stellwagen and Malenka, 2006). We found a significant increase in mEPSC inter-event interval (consistent with a decrease in frequency) in deprived animals and deprived animals that were injected with the TNF-α inhibitor (Figures S1Q–S1T).

Synaptic scaling is also associated with an increase in the AMPAR subunit GluA2 (Gainey et al., 2009), which is facilitated

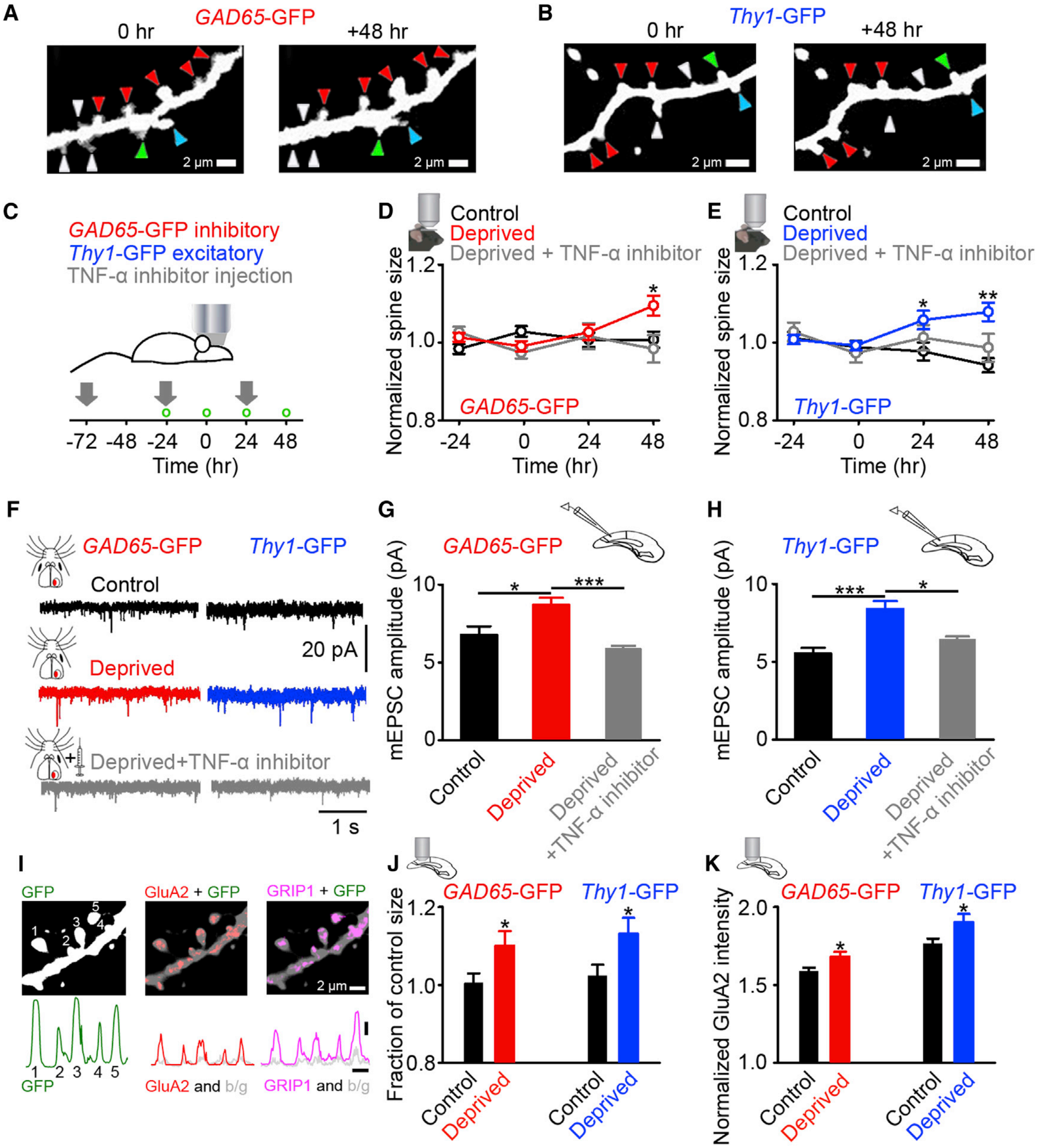

Figure 1. Spine Size Changes and Synaptic Scaling in Inhibitory and Excitatory Neurons

(A and B) Example *in vivo* image projections. Arrowheads show spines that increase (red), decrease (blue), stay the same size (green), or are lost (white).

(C) Experimental timeline. Enucleation occurs immediately after imaging at 0 hr. Gray arrows indicate time of TNF-α inhibitor injections; green circles indicate time of *in vivo* imaging.

(D and E) Spine size normalized to baseline (average of time points -24 and 0) for individual spines in control (black), deprived (red/blue), or deprived with the TNF-α inhibitor (gray) for inhibitory (D) or excitatory (E) neurons. Asterisks denote statistics from one-way repeated-measures ANOVA (see Table S1).

(F–H) Example mEPSC recordings (F) or average mEPSC amplitude per cell 48 hr after enucleation (red/blue), enucleation with the TNF-α inhibitor (gray), or control (black).

(I) (Top) Dendritic section from an excitatory neuron 48 hr after deprivation with immunohistochemistry against GFP (left), GluA2 (middle), and GRIP1 (right). (Bottom) Line trace of fluorescence intensity and the background measured by rotating the individual fluorescence images by 90° (gray). Scale bars, 2 μm and 25 intensity units.

(J and K) Spine size normalized to average control spine size (J) and fluorescence intensity of GluA2 in spines that co-localized with GRIP1 normalized to the background GluA2 fluorescence (K, see STAR Methods and Figures S1U–S1Z) for branches from either inhibitory (red) or excitatory (blue) neurons 48 hr after enucleation or control (black). (Insets) Mouse with objective is *in vivo* imaging experiment, slice with objective is *in vitro* imaging experiment, and slice with electrode is *in vitro* electrophysiology experiment. *$p < 0.05$; **$p < 0.01$; ***$p < 0.001$. For statistical comparisons and n values, see Table S1. Error bars, mean and SEM. Crossing axons have been removed from images for clarity.

See also Figure S1 and Table S1.

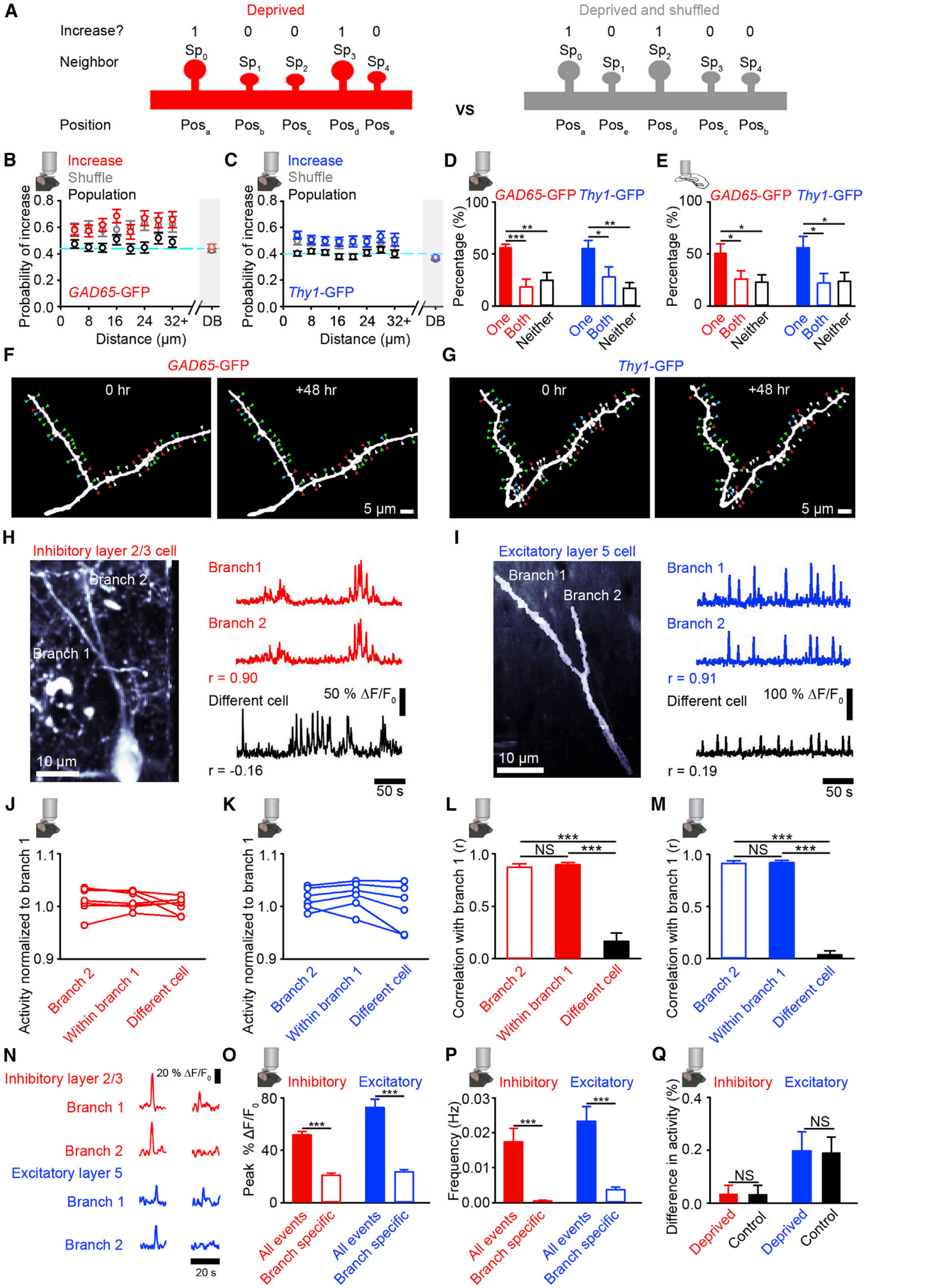

(legend on next page)

by interactions with the AMPAR-binding protein Glutamate Receptor Interacting Protein 1 (GRIP1) (Gainey et al., 2015). To examine this molecular signature of synaptic scaling, we again prepared brain slices from mice 48 hr after deprivation and performed immunohistochemistry against GFP, GluA2, and GRIP1 (Figures 1I and S1U–S1Y). Spine size (Figure 1J), the intensity of GluA2 that is co-localized with GRIP1 in spines (Figure 1K), and the GluA2 intensity per unit spine size (Figure S1Z) increased following deprivation in both inhibitory and excitatory cells, consistent with measures of synaptic scaling (Gainey et al., 2009, 2015). Taken together, these results suggest that the observed net increases in spine size are likely due to synaptic scaling, not Hebbian mechanisms, in both inhibitory and excitatory neurons.

While synaptic scaling is generally thought to occur cell-wide (Turrigiano, 2012), it has been demonstrated to be inducible locally at individual dendritic branches in reduced preparations (Sutton et al., 2006). It is still unclear, however, if synaptic scaling is compartmentalized to individual dendritic branches *in vivo*. Given the evidence that *in vivo* spine size changes reflect a TNF-α-dependent scaling-like process, we used spine size increases as a proxy for synaptic scaling to investigate the spatial scale of this process *in vivo*. To this end, we analyzed the spatial clustering of spine size changes following deprivation. We first quantified if there is clustering of increasing spines within a dendritic branch (see STAR Methods) and found no evidence for spatial clustering more local than branch-wide in either inhibitory (Figures 2A, 2B, S2A, S2C, and S2E) or excitatory (Figures 2A, 2C, S2B, S2D, and S2F) neurons. We then expanded the spatial scale of our analysis to determine if all branches on a cell undergo spine size increases. We found that spines on the same branch as an increasing spine were more likely to increase in size than the population average (Figures 2B and 2C), while spines on a different branch (DB) of the same cell were not (Figures 2B and 2C). We then calculated whether each dendritic branch underwent average increases in spine size or not (see STAR Methods for criteria for "increasing branches"). Overall, we found that roughly half of all branches in both inhibitory (18/31 branches, or 58%) and excitatory (12/24 branches, or 50%) neurons underwent increases in spine size (see STAR Methods). This proportion was greater than both the fraction of branches that we would expect to increase by chance given the number of increasing spines (see STAR Methods; inhibitory cell branches, 30% ± 1%; excitatory cell branches, 31% ± 1%), and those we measured in control animals (inhibitory cell branches, 11/40 or 28%; excitatory cell branches, 7/34 or 21%). Within individual cells, we found that pairs of branches on the same cell did not always both undergo increases in spine size following enucleation in either inhibitory or excitatory neurons (Figure 2D). We confirmed these *in vivo* findings by imaging dendritic branches in slices prepared from mice that had previously undergone deprivation, showing that pairs of branches on the same cell do not always both have increases in spine size (Figure 2E). In these slices, we were able to reconstruct a larger percentage of the dendritic arbor and found that branches with spines that increase in size occurred across all branch orders on the dendritic tree (Figures S2G and S2H). These results suggest that increases in spine size do not occur uniformly across all dendritic branches of a given neuron. Instead, spines that increase in size were preferentially located on a subset of the branches in the dendritic tree. This result seemed inconsistent with the observation of multiplicative synaptic scaling from the electrophysiology data (Figures S2I, S2K, S2M, and S2O), so we used established methods (Kim et al., 2012, see STAR Methods) to determine if multiplicative scaling could occur with only 50% of inputs increasing in size. Consistent with the *in vivo* imaging results, we found that multiplicative scaling of only 50% of the control mEPSC amplitude distribution gave a better fit to

Figure 2. Branch-Specific spine Size Changes *In Vivo*

(A) Whether neighbors ($Sp_{1...n}$) of spine, Sp_0, increase in size (1 increasing, 0 not increasing) is calculated for distances from Sp_0 (red). Shuffled (gray) versions of the same dendrite are created by randomly assigning the spatial positions of the spines ($Pos_{a...d}$) as a comparison to the experimental dendrites.

(B and C) Cluster analysis for inhibitory (B) and excitatory (C) neurons. The fraction of spines, at different distances (4 μm bins) from a spine (Sp_0) in deprived (red/blue) animals, that increase in size (at least 1.1 times larger than baseline at 48 hr post-enucleation). Data are plotted when Sp_0 is a spine that increases (red/blue), is any spine from the entire population (black), and where the spatial position of neighboring spines for each increasing Sp_0 is randomly shuffled (gray, Figure 2A). DB is the probability of an increasing spine on the same cell, but a different branch. Cyan dashed line depicts proportion of all spines increasing.

(D and E) Percentage of branch pairs, where average spine size is increased relative to baseline (see STAR Methods for criteria) for *in vivo* (D) or increased relative to average control size for *in vitro* (E) on one branch (red/blue filled), both branches (red/blue open), or neither branch (black, open). Percentages are averaged across cells.

(F and G) Example *in vivo* images of a branch pair. Arrowheads show spines that increase (red), decrease (blue), stay the same size (green), or are lost (white) after enucleation. In both (F) and (G), the right branch has an average increase in spine size, while the left branch does not. Crossing axons have been removed for clarity.

(H and I) (Left) Image of a dendritic branchpoint on a cell expressing GCaMP6f. Image in (I) is side projected. (Right) Change in fluorescence signals (%$\Delta F/F_0$) for labeled branches (red/blue) or from a dendrite in the same imaging region, but on a different cell (black).

(J–M) Activity levels (integral of the %$\Delta F/F_0$ signal) normalized to the overall activity in branch 1 (J and K) or the correlation coefficient calculated with branch 1 (L and M), for a dendritic branch sharing a branchpoint (branch 2), for a neighboring region 10 μm apart on the same branch (within branch 1) and for a branch in the same imaging region but on a different cell (different cell) in inhibitory neurons 24 hr post-enucleation (J and L) or excitatory neurons 4 hr post-enucleation (K and M).

(N) Example calcium signals (%$\Delta F/F_0$) from branch pairs showing global (left) and branch-specific calcium events (right).

(O and P) Average peak amplitude (O) or average frequency (P) of all dendritic calcium events and branch-specific calcium events.

(Q) Percentage difference in total calcium activity attributable to branch specific events (see STAR Methods) between branches in branch pairs. (Insets) Mouse with objective is *in vivo* imaging experiment; slice with objective is *in vitro* imaging experiment. NS, no significance; *$p < 0.05$; **$p < 0.01$; ***$p < 0.001$. For statistical comparisons and n values, see Table S2. Error bars, mean and SEM.

See also Figure S2 and Table S2.

the deprived distribution than scaling 100% of the control distribution (Figures S2I–S2P).

The engagement of homeostatic synaptic strengthening mechanisms, including synaptic scaling, is thought to depend on strong changes in supra-threshold postsynaptic activity (Ibata et al., 2008). To test if differences in supra-threshold dendritic activity between branches on the same cell could explain the branch-specific changes in spine size that we observed *in vivo*, we expressed GCaMP6f (Chen et al., 2013) in (NPY-positive) inhibitory or, in a separate set of animals, layer 5 excitatory cells (see STAR Methods). We then measured dendritic calcium transients, as a proxy for suprathreshold activity (back-propagating action potentials and dendritic spikes) in awake head-fixed mice, which were free to run on a spherical treadmill with visual stimulation coupled to the mouse's movement. In dendritic branches (Figures 2H and 2I) that shared a branchpoint (as with the structural imaging data in Figures 2D and 2E) located in layer 2/3, we examined calcium transients after enucleation, but before we observed spine size increases (see STAR Methods). We compared the activity in a dendritic branch with (1) activity in another branch that shared a branchpoint, (2) activity in a neighboring region 10 μm away on the same dendritic branch (which provides an approximation of the within-branch signal variability over a spatial scale in which we measured all of the structural imaging branches and do not observe differences in spine size changes, see Figures 2B and 2C), or (3) activity in a branch from a different cell in the same imaging region. Activity levels (measured as the integral of the $\Delta F/F_0$ signal in the imaging session) were similar in all conditions (Figures 2J and 2K). The variation in activity patterns (measured as the correlation and mutual information) between branch pairs was indistinguishable from that measured within a single dendritic branch (Figures 2L, 2M, S2Q, and S2R) but was very different from branches of other neurons in the same imaging field of view for both cell types (Figures 2L, 2M, S2Q, and S2R). We next examined branch-specific calcium events that occurred in only one of the two dendritic branches (Figure 2N; see STAR Methods). We measured the peak amplitude (Figure 2O) and frequency (Figure 2P) of branch-specific events and found that these events accounted for a small fraction (<1%) of the overall activity in the branches (Figure S2S). Specifically, the activity differences between the branches as a result of these branch-specific calcium events was very small (on the order of 0.20%) and was also similar to the difference in activity between branch pairs attributable to branch-specific events in control animals (Figure 2Q). Together, these results indicate that between dendritic branchpoint pairs, there are not strong differences in suprathreshold activity that are typically associated with the induction of synaptic scaling (Ibata et al., 2008).

Given that we did not see large differences in postsynaptic activity between branchpoint pairs, we next examined whether there were clear differences in the total input to branches by measuring spine density in the chronic *in vivo* imaging data. We found that dendritic branches with spines that underwent increases in size following deprivation also lost more spines following enucleation. They therefore had a lower spine density after enucleation than branches whose spines stayed the same size or decreased following deprivation (Figures 3A–3C and S3A). It is important to note that the observed effect is not simply that small spines are lost and the absolute spine sizes on the branches are larger after deprivation. Instead, spines on increasing branches get larger relative to their individual sizes prior to deprivation, which is paralleled by spine loss within that dendritic branch. This decrease in spine density was largely a function of spine loss, as we measured no significant difference in new spine formation between control and deprived animals (percentage new spines: inhibitory, control 3.6% versus deprived 3.9%, p = 0.493; excitatory, control 9.3% versus deprived 7.7%, p = 0.993, Chi-square test). Decreased spine density occurred in both inhibitory and excitatory cells (Figures 3A–3C, S3A, and S3B), but we found no evidence that increasing branches depend on cortical depth (Figure S3C), initial spine size (Figure S3D), or initial spine density (Figure S3E). This decrease in density was observed across a number of spine size increase thresholds (Figure S3F). We also observed no spatial clustering within dendritic branches of spine loss (Figures S3G and S3H) and found that dendrite width did not change or alter spine size measures following enucleation (Figures S3I–S3N).

We next examined the relative timing of the observed changes by chronically measuring spine size and density following monocular enucleation with *in vivo* two-photon imaging. Because these increases in spine size happen more rapidly than 24 hr in excitatory cells (but not inhibitory cells), we repeatedly measured the same dendritic branches over a period of 8 hr after enucleation for excitatory cells and over 48 hr for inhibitory neurons. We found that spine density decreases prior to increases in spine size in both inhibitory (Figure 3D) and excitatory (Figure 3E) neurons.

We then examined if there was a relationship between spine size and spine loss within a dendritic branch *in vivo*. Following enucleation, we found a negative correlation between the normalized spine density and the normalized size of the remaining spines on that branch for both inhibitory (Figure 3F) and excitatory (Figure 3G) neurons. Therefore, on dendritic branches that lose more spines after deprivation, the remaining spines undergo greater size increases (Figures 3F–3H), but only following enucleation (Figures S3O and S3P). We then examined this inverse relationship between spine size and spine density in slices we prepared from deprived mice. In these data, we were able to examine branches across the entire dendritic tree, rather than just the distal branches. Across dendrites for all branch orders (Figures S3Q and S3R), spine density was lower than control in the dendritic branches whose average spine size was significantly higher than control (Figures 3I and S3B). In both inhibitory (Figure 3J) and excitatory (Figure 3K) neurons, spine density was inversely correlated with spine size. These data further support the results observed with repeated *in vivo* imaging. This relationship between spine size and density was absent in deprived mice injected with the TNF-α inhibitor, where spine loss still occurred (Figures 3L, 3M, and S3S–S3V), but there was no spine size increase (Figure 3N). Taken together, these results suggest that increases in spine size occur in a dendritic branch-specific manner and are correlated with the degree of preceding spine loss on that branch.

In comparison to the traditional view of global synaptic scaling, with branch-specific synaptic scaling, homeostatic processes

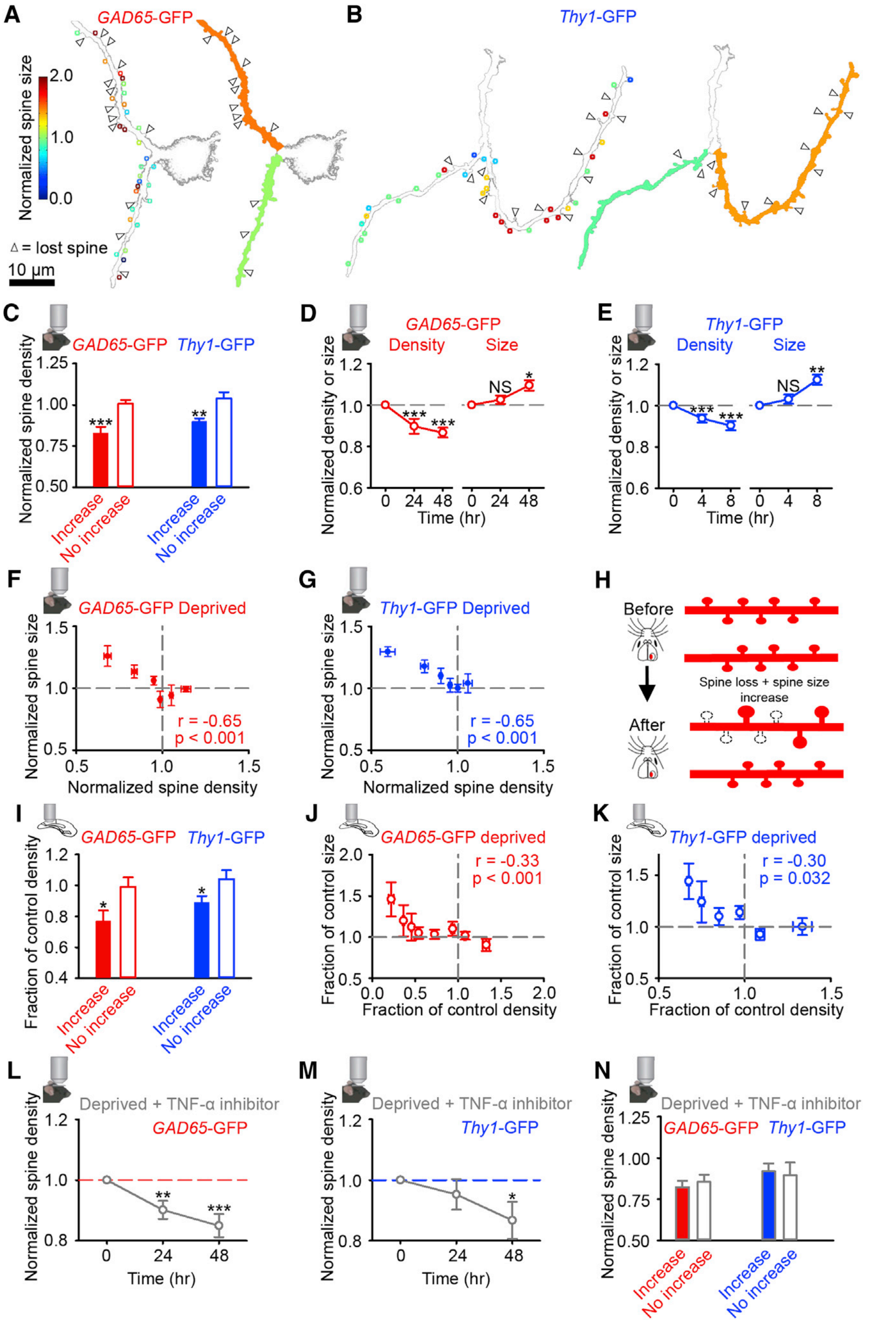

Figure 3. Relationships between Spine Loss and Increases in Spine Size within Dendritic Branches

(A and B) Example branches showing spine loss (arrowhead) and stable spine size (circles) after deprivation. Color scale shows spine size 48 hr post-enucleation normalized to baseline for individual spines (left). The average spine size change over a branch (right) is shown by the color of the filled branch and corresponds to scale in (A).

(C) Spine density 48 hr post-enucleation normalized to baseline for individual branches whose spines increase in size or do not increase relative to their individual baseline after enucleation (see STAR Methods).

(D and E) Normalized spine size and spine density after enucleation for inhibitory (D) and excitatory (E) neurons.

(F and G) Spine density versus average spine size normalized to baseline. Data taken at 48 hr (F) and 8 hr (G) after enucleation and normalized to baseline values for each branch (density) and individual spines (size). Normalized size change is then averaged across the dendritic branch.

(H) Schematic showing the observed relationship between spine loss and spine size change before (top) and after (bottom) enucleation. Black dashed lines show lost spines.

(I) Spine density (as a fraction of the average control value) for increasing and non-increasing spine size branches (see STAR Methods) in slices prepared 48 hr after enucleation.

(J and K) Spine density versus spine size (as a fraction of the average control values for branches (density) and individual spines (size), and size is then averaged across the branch) in slices prepared 48 hr after enucleation.

(L and M) Spine density normalized per branch to baseline for dendritic branches in deprived animals with the TNF-α inhibitor.

(N) Spine density in deprived animals with the TNF-α inhibitor 48 hr post-enucleation normalized to baseline for individual branches whose spines increase (filled) or do not increase (open) in size relative to their individual baseline after enucleation. (Insets) Mouse with objective is *in vivo* imaging experiment, slice with objective is *in vitro* imaging experiment. NS, no significance; *p < 0.05; **p < 0.01; ***p < 0.001. For statistical comparisons and n values, see Table S3. Error bars, mean and SEM.

See also Figure S3 and Table S3.

are most prominent on dendritic branches that have undergone input loss. A simple consequence of this effect is that activity can be locally restored in deprived branches, without disturbing the existing input-output relationships at the unaffected branches. However, the relative weights of the synapses across the cell are perturbed, since only a subset of synapses are strengthened. We investigated whether there are neural processing benefits to spatially restricted synaptic scaling in comparison to global scaling. We developed a model to examine the effect of branch-specific versus global synaptic scaling on the mutual information between input ensembles and spiking output. To do this, we used an abstract two-layer "neural network" model (see STAR Methods; Poirazi et al., 2003; Figure 4A) that consists of inputs that are summed and passed through non-linear dendritic compartments. These dendritic outputs are then summed and passed through an additional non-linearity at the soma.

To simulate the *in vivo* findings, we then eliminated a subset of the inputs (on average 10%) to 50% of dendritic branches and subsequently either (1) scaled synaptic weights on individual branches depending on their degree of individual input loss (branch specific) or (2) scaled synaptic weights equally across all branches by an amount proportional to the total spine loss

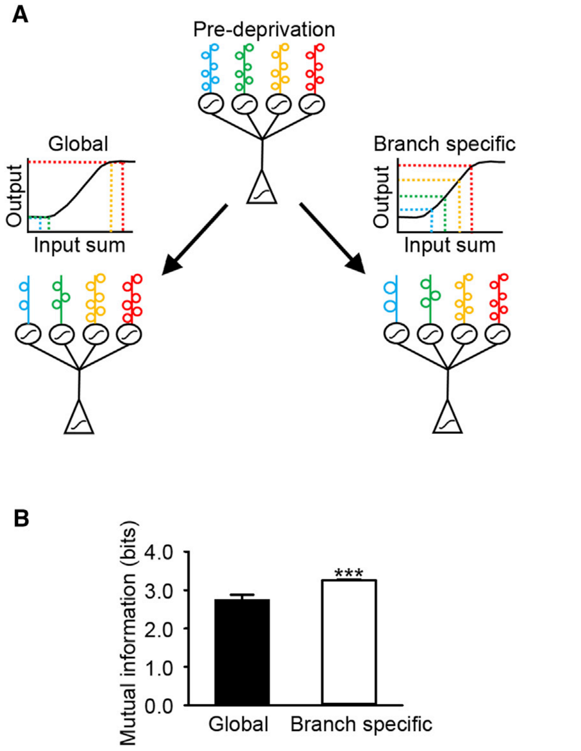

Figure 4. Model Comparing Mutual Information in Conditions of Branch-Specific and Global Synaptic Scaling

(A) Schematic of model. Pre-deprivation: model architecture with synaptic weights as spines (colored open circles) on dendritic branches (colored vertical lines at top of schematic). The weights of the randomly chosen subset of activated inputs are summed and passed through a dendritic sigmoidal function (black circles). Then the individual branches' activities are summed and passed through a somatic sigmoidal function (black triangle). (Bottom left) Global scaling following spine loss. (Bottom right) Branch-specific scaling following the same spine loss. Sigmoidal plots above the model represent the dendritic branch sigmoid, showing examples of summed dendritic inputs (colored vertical lines on sigmoid) translating to an output (corresponding color, horizontal lines on sigmoid).

(B) Mutual information values (global versus branch specific, $p < 0.001$, t test) for simulations. ***$p < 0.001$. Error bars, mean and SD.

in the cell (global) (Figure 4A). We measured the response of the cell (somatic output) to the activation of a fraction of randomly chosen inputs (see STAR Methods). We found that when weights were normalized within a branch rather than globally, the mutual information (Figure 4B) between the stimulus (i.e., the specific pattern of inputs that were activated) and the cell's output was higher, suggesting that the neuron has a greater capacity for information after branch-specific scaling. These results are due to a combination of two effects. First, with local scaling, unaffected branches are not modified, so their input-output relationships are unaltered, as described above. Second, in the global scaling condition (Figure 4A, global), more branches have larger outputs (due to increases in input size without input loss on some branches), so their input sum moves onto the non-linear high plateau section of the sigmoid. Therefore, several different inputs (Figure 4A, global top, vertical yellow and red dashed lines) result in the same output (Figure 4A, global top, horizontal red dashed line) and are thus indistinguishable. Also with global scaling, several branches are very low in summed input due to spine loss and limited scaling (Figure 4A, global top, vertical blue and green dashed lines). Their input sum is in the lower non-linear part of the sigmoid, and they therefore map to the same output (Figure 4A, global top, horizontal green dashed line). In branch-specific scaling, branches remain in the linear component of the sigmoid and thus have distinguishable outputs (Figure 4A, branch specific), resulting in higher mutual information between the inputs and outputs (Figure 4B). Thus, this local implementation of synaptic scaling provides a mechanism by which activity levels can be adjusted to prevent extreme firing rates, with increases to the information processing capabilities of the neuron relative to global scaling.

DISCUSSION

Branch-Specific Increases in Spine Size

We find that spine size increases following deprivation are TNF-α dependent and occur in parallel with classical signatures of synaptic scaling (mEPSC amplitude increase following decreased sensory activity and increased GluA2 co-localized with GRIP1 in spines). The spatial scale of synaptic scaling *in vivo* was previously unclear, but there had been indications from reduced preparations that mechanisms associated with scaling can be implemented more locally than cell-wide (Ju et al., 2004; Sutton et al., 2006). We found that increases in spine size occurred on dendritic branches that had undergone recent spine loss in a subset of both inhibitory and excitatory neurons. Given that this effect occurs across multiple cell types and layers, it may reflect a general phenomenon of homeostatic synaptic compensation following deprivation; however, it is important to note that laminar differences have been reported in homeostatic plasticity paradigms (Desai et al., 2002), and therefore the mechanisms underlying this phenomenon may be layer specific. Previous work has suggested that plasticity can be implemented locally in dendritic branches (Losonczy et al., 2008; Makara et al., 2009; Sutton et al., 2006) and that there is synaptic weight homeostasis (Bourne and Harris, 2011), where the overall sum of synaptic area on a dendritic branch is constant across dendritic branches and cells. Together with these previous studies, our results provide further evidence for the dendritic branch as a key processing unit in the brain (Branco and Häusser, 2010).

Synaptic Scaling in Inhibitory Neurons

While synaptic scaling has previously been shown to occur in inhibitory neurons in reduced preparations (Hartman et al., 2006), here we demonstrate that synaptic scaling occurs following *in vivo* deprivation in what are likely NPY-positive inhibitory neurons measured via an increase in mEPSC amplitudes. NPY-positive inhibitory neurons have been implicated in inhibiting seizure-like events in cortex and hippocampus (Baraban et al., 1997; Bijak, 2000). Thus, homeostatic regulation of their activity levels may be important for preventing runaway excitation. More generally, inhibitory neurons are not only instrumental in regulating network activity levels but also play a role in sharpening tuning curves (Lee et al., 2012; Mao et al., 2012; Tremblay

et al., 2016) and modulating the timing of neural responses. Thus, how and when inhibitory neurons engage homeostatic mechanisms or change their firing rates will influence the overall network activity of excitatory cells.

In the present study, we only examine synaptic scaling, but the resulting activity levels of inhibitory neurons after deprivation will reflect the interactions between multiple homeostatic mechanisms—synaptic scaling, changes in intrinsic excitability, and altering the balance between excitation and inhibition. We have previously reported that on average inhibitory neurons in layer 2/3 of visual cortex undergo an extended (at least 48 hr) reduction in activity following deprivation (Barnes et al., 2015); however, within the population, we observed a wide range of activity responses following deprivation, from inhibitory cells that became functionally silent to those that underwent complete recovery of activity or even became more active after deprivation. We previously speculated that the variability in responses could be inhibitory cell type specific (Barnes et al., 2015), but to date the relationship between homeostatic activity profiles and cell type is still unknown. Different inhibitory subtypes may play different roles in homeostatic plasticity, and therefore our results regarding synaptic scaling could be specific to the inhibitory subtype studied here. If synaptic scaling would occur in many inhibitory subtypes, for cells that show a reduction of activity, scaling alone may not be sufficient to fully restore activity levels to pre-deprivation set points (Hengen et al., 2016). This interpretation would suggest a balancing role for inhibitory cells in network homeostasis after sensory deprivation, where activity levels in inhibitory neurons remain at moderate levels, sufficient to prevent runaway excitation if necessary, but still low enough to promote overall recovery of activity in the excitatory cells. Elucidating activity profiles and cell-intrinsic and synaptic plasticity mechanisms for specific inhibitory subtypes will be essential for understanding how changes in inhibitory cell activity facilitate network plasticity following input loss.

Potential Mechanisms

We demonstrated that the branch-specific increases in spine size are dependent on TNF-α, which is thought to be released from glia in response to a decrease in glutamate and to facilitate the induction of synaptic scaling (Stellwagen and Malenka, 2006). Given that glial processes are adjacent to individual or small groups of synapses (Lin and Bergles, 2004), one possibility is that focal TNF-α release initiates plasticity mechanisms that are spatially mediated by factors intrinsic to the dendrite. These could include molecules with the potential to work at the spatial scale of a dendritic branch. Potential candidates would include retinoic acid (Aoto et al., 2008) and brain-derived neurotrophic factor (BDNF) (Rutherford et al., 1998), among others. Our results are also consistent with work showing that the diffusion of postsynaptic components is spatially confined within individual dendritic branches (Cui-Wang et al., 2012). Thus, the branch-specific nature of our findings may be attributable to postsynaptic plasticity mechanisms, rather than coordinated TNF-α release at numerous synapses across the dendrite.

Given that we observe a decrease in mEPSC frequency, we cannot rule out that presynaptic changes may also contribute to our observed effects, particularly in the initiation of spine loss. The spine loss we observe is not dependent on TNF-α and thus may be mediated by Hebbian-like mechanisms. Previous work has shown clustered spine formation following learning paradigms (Fu et al., 2012), which may be facilitated by local molecular mechanisms (Harvey et al., 2008) associated with Hebbian plasticity induction. Here, we did not find evidence for clustered spine loss following deprivation (Figures S3G and S3H). Thus, while Hebbian depression of synapses may be facilitated by local activity interactions (Winnubst et al., 2015), deprivation-induced spine loss itself may be more spatially widespread. Previous work in hippocampus has suggested that following potentiation of groups of spines on a dendritic branch, neighboring spines that were not co-stimulated are weakened (Oh et al., 2015). In our study, we instead find that spine loss precedes spine size increases. Given that in the study of Oh et al. (2015) spine size increase is induced by high frequency stimulation and not a sensory deprivation-induced protocol, the precise mechanisms may be different, but the study does suggest that the local balance between spines that share a dendritic branch may apply more generally and could act through heterosynaptic mechanisms (Keck et al., 2017). The exact combination of molecular mechanisms underlying our branch-specific phenomenon beyond the dependence on TNF-α release will require future investigation.

We observe that dendritic branches on the same cell do not have the same degree of changes in spine size. While our experiments using the calcium indicator GCaMP6f are likely dominated by suprathreshold activity, we did not see dramatic differences in activity levels or patterns between two branches sharing a branchpoint, and we observed very few dendritic branch-specific events that would be typically associated with dendritic calcium spikes (Cichon and Gan, 2015). These results are consistent with previous calcium imaging studies that suggest branch-specific calcium events are more prominent in motor cortex (Cichon and Gan, 2015) than in sensory cortices (Xu et al., 2012). Synaptic scaling is generally thought to require large changes (greater than 20%) in activity levels (Ibata et al., 2008; Turrigiano, 2011), and we only detect differences in neighboring dendritic activity on the order of 0.2% or less (Figure 2Q). We also observed that the proportional relationship between spine size changes and spine loss was only present in deprived animals (Figures 3F, 3G, S3O, and S3P), suggesting that net spine size increases may require input loss or an overall reduction in activity. In line with previous studies (Fong et al., 2015; Sutton et al., 2006), one possible interpretation of our results is that more subtle changes in subthreshold activity associated with input loss may be enough to induce synaptic scaling differentially among dendritic branches in the same cell. This idea is consistent with previous work that indicates functional changes at the level of the dendrite are sufficient to induce plasticity, independent of the global changes in activity levels and patterns (Losonczy et al., 2008; Makara et al., 2009; Sutton et al., 2006).

Consequences for the Network

One of the important properties of synaptic scaling is that by changing the strength of the synapses while maintaining their relative weights, the input-output relationships that were developed prior to deprivation through experience and learning are

maintained. We used simulations to compare the effects of global scaling with branch-specific scaling on the mutual information between the inputs and outputs of the neuron. We found that mutual information was greater when scaling occurred locally within a branch, suggesting that information capacity is not maximized with global scaling. This effect results from including an experimentally reported dendritic non-linearity (Branco and Häusser, 2010, 2011; Poirazi et al., 2003). With global scaling, branches that have not lost spines are still scaled, increasing the overall structural input to the dendrite. Additionally, on branches that have lost many spines, the maintained spines are only moderately increased in strength because all spines on the cell are scaled by an equal fraction. Thus, in the global scaling condition, a fraction of dendritic branches has either very high or very low levels of input because the change in synaptic weights is not proportional to local spine loss. Because of the dendritic non-linearity (Figure 4A, global), these outlier branches will end up in the extreme ends of the sigmoid summation (the plateaus) and thus will have an altered input-output relationship. Alternatively, with branch-specific scaling, the maintained spines are scaled in proportion to the degree of local spine loss. As a result, the dendrites are less likely to have such extreme input levels because the total spine weight is balanced in individual branches (Figure 4A, branch specific). A potential benefit of global scaling is that the relative weights of all inputs across the cell are maintained, since they are all changed by the same fraction. One consequence of branch-specific scaling is that the relative weights of individual inputs are altered across branches, since only affected dendrites undergo scaling and thus their individual inputs are increased relative to the population. The consequences of this for neural coding are currently unknown. Our simulations indicate that branch-specific scaling is associated with an increase in mutual information relative to global scaling. Thus, the compartmentalized form of branch-specific scaling may better preserve input-output relationships in neurons with dendritic non-linearities.

Mouse visual cortex is known to be multimodal, showing neural responses to auditory (Ibrahim et al., 2016; Iurilli et al., 2012), motor (Andermann et al., 2011; Ayaz et al., 2013; Keller et al., 2012; Niell and Stryker, 2010; Pakan et al., 2016; Saleem et al., 2013; Zmarz and Keller, 2016), and somatosensory stimuli (Yoshitake et al., 2013), as well as contextual signals (Attinger et al., 2017; Fiser et al., 2016; Roth et al., 2016; Tohmi et al., 2014). Given recent evidence for inputs with similar properties clustering within a dendritic branch (Iacaruso et al., 2017; Wilson et al., 2016), alterations to the animal's environment related to a particular stimulus type may lead to drastic activity changes in some branches where inputs related to that stimulus cluster, but not others that have inputs for different types of stimuli. Local compensation of synapses (and in theory activity) prevents any particular stimuli from dominating the output activity levels and patterns for any extended period of time. Additionally, adjusting activity locally in dendritic branches will likely result in activity levels being maintained cell- and network-wide. Overall, branch-specific homeostatic changes provide a mechanism by which activity levels can be regulated without substantial disruption to the existing circuitry that is unaffected by sensory deprivation.

STAR★METHODS

Detailed methods are provided in the online version of this paper and include the following:

- KEY RESOURCES TABLE
- CONTACT FOR REAGENT AND RESOURCE SHARING
- EXPERIMENTAL MODEL AND SUBJECT DETAILS
  - Animals
- METHOD DETAILS
  - Surgery
  - Immunohistochemistry
  - Electrophysiology
  - Model
- QUANTIFICATION AND STATISTICAL ANALYSIS
  - Functional imaging and analysis
  - Structural imaging and analysis
  - Immunohistochemistry image analysis
  - Electrophysiology analysis
  - Statistics
- DATA AND SOFTWARE AVAILABILITY
  - Code availability

SUPPLEMENTAL INFORMATION

Supplemental Information includes three figures and three tables and can be found with this article at https://doi.org/10.1016/j.neuron.2017.09.052.

AUTHOR CONTRIBUTIONS

S.J.B., E.F., R.I.J., C.C., G.B.K., and T.K. performed experiments, analyzed data, and wrote the manuscript. F.E. and G.S. engineered the *GAD65*-GFP mouse line.

ACKNOWLEDGMENTS

We thank Laura Andreae, Claire Cheetham, Sonja B. Hofer, Nicholas A. Lesica, and members of the Keller lab for comments on the manuscript and helpful discussions, and Rosanna P. Sammons, David Elliott, and Anton Gauert for assistance with analysis. This work was supported by the Medical Research Council (S.J.B., R.I.J., and T.K.), the European Research Council (S.J.B., E.F., T.K., and C.C.), The Edmond J. Safra Foundation (S.J.B.), the Royal Society (T.K.), the Wellcome Trust (T.K. and C.C.), the Novartis Research Foundation (G.B.K.), and the Swiss National Science Foundation (G.B.K.). XPRo1595 was provided by Xencor, Inc.

Received: April 3, 2017
Revised: August 16, 2017
Accepted: September 27, 2017
Published: October 26, 2017

REFERENCES

Andermann, M.L., Kerlin, A.M., Roumis, D.K., Glickfeld, L.L., and Reid, R.C. (2011). Functional specialization of mouse higher visual cortical areas. Neuron *72*, 1025–1039.

Aoto, J., Nam, C.I., Poon, M.M., Ting, P., and Chen, L. (2008). Synaptic signaling by all-trans retinoic acid in homeostatic synaptic plasticity. Neuron *60*, 308–320.

Attinger, A., Wang, B., and Keller, G.B. (2017). Visuomotor coupling shapes the functional development of mouse visual cortex. Cell *169*, 1291–1302.e14.

Ayaz, A., Saleem, A.B., Schölvinck, M.L., and Carandini, M. (2013). Locomotion controls spatial integration in mouse visual cortex. Curr. Biol. *23*, 890–894.

Baraban, S.C., Hollopeter, G., Erickson, J.C., Schwartzkroin, P.A., and Palmiter, R.D. (1997). Knock-out mice reveal a critical antiepileptic role for neuropeptide Y. J. Neurosci. *17*, 8927–8936.

Barnes, S.J., Sammons, R.P., Jacobsen, R.I., Mackie, J., Keller, G.B., and Keck, T. (2015). Subnetwork-specific homeostatic plasticity in mouse visual cortex in vivo. Neuron *86*, 1290–1303.

Bijak, M. (2000). Neuropeptide Y reduces epileptiform discharges and excitatory synaptic transmission in rat frontal cortex in vitro. Neuroscience *96*, 487–494.

Bourne, J.N., and Harris, K.M. (2011). Coordination of size and number of excitatory and inhibitory synapses results in a balanced structural plasticity along mature hippocampal CA1 dendrites during LTP. Hippocampus *21*, 354–373.

Branco, T., and Häusser, M. (2010). The single dendritic branch as a fundamental functional unit in the nervous system. Curr. Opin. Neurobiol. *20*, 494–502.

Branco, T., and Häusser, M. (2011). Synaptic integration gradients in single cortical pyramidal cell dendrites. Neuron *69*, 885–892.

Cheetham, C.E.J., Barnes, S.J., Albieri, G., Knott, G.W., and Finnerty, G.T. (2012). Pansynaptic enlargement at adult cortical connections strengthened by experience. Cereb. Cortex *24*, 521–531.

Chen, T.-W., Wardill, T.J., Sun, Y., Pulver, S.R., Renninger, S.L., Baohan, A., Schreiter, E.R., Kerr, R.A., Orger, M.B., Jayaraman, V., et al. (2013). Ultrasensitive fluorescent proteins for imaging neuronal activity. Nature *499*, 295–300.

Cichon, J., and Gan, W.-B. (2015). Branch-specific dendritic Ca(2+) spikes cause persistent synaptic plasticity. Nature *520*, 180–185.

Cui-Wang, T., Hanus, C., Cui, T., Helton, T., Bourne, J., Watson, D., Harris, K.M., and Ehlers, M.D. (2012). Local zones of endoplasmic reticulum complexity confine cargo in neuronal dendrites. Cell *148*, 309–321.

Desai, N.S., Cudmore, R.H., Nelson, S.B., and Turrigiano, G.G. (2002). Critical periods for experience-dependent synaptic scaling in visual cortex. Nat. Neurosci. *5*, 783–789.

Dombeck, D.A., Khabbaz, A.N., Collman, F., Adelman, T.L., and Tank, D.W. (2007). Imaging large-scale neural activity with cellular resolution in awake, mobile mice. Neuron *56*, 43–57.

Feldman, D.E. (2000). Timing-based LTP and LTD at vertical inputs to layer II/III pyramidal cells in rat barrel cortex. Neuron *27*, 45–56.

Feng, G., Mellor, R.H., Bernstein, M., Keller-Peck, C., Nguyen, Q.T., Wallace, M., Nerbonne, J.M., Lichtman, J.W., and Sanes, J.R. (2000). Imaging neuronal subsets in transgenic mice expressing multiple spectral variants of GFP. Neuron *28*, 41–51.

Fiser, A., Mahringer, D., Oyibo, H.K., Petersen, A.V., Leinweber, M., and Keller, G.B. (2016). Experience-dependent spatial expectations in mouse visual cortex. Nat. Neurosci. *19*, 1658–1664.

Fong, M.F., Newman, J.P., Potter, S.M., and Wenner, P. (2015). Upward synaptic scaling is dependent on neurotransmission rather than spiking. Nat. Commun. *6*, 6339.

Fu, M., Yu, X., Lu, J., and Zuo, Y. (2012). Repetitive motor learning induces coordinated formation of clustered dendritic spines in vivo. Nature *483*, 92–95.

Gainey, M.A., Hurvitz-Wolff, J.R., Lambo, M.E., and Turrigiano, G.G. (2009). Synaptic scaling requires the GluR2 subunit of the AMPA receptor. J. Neurosci. *29*, 6479–6489.

Gainey, M.A., Tatavarty, V., Nahmani, M., Lin, H., and Turrigiano, G.G. (2015). Activity-dependent synaptic GRIP1 accumulation drives synaptic scaling up in response to action potential blockade. Proc. Natl. Acad. Sci. USA *112*, E3590–E3599.

Gerfen, C.R., Paletzki, R., and Heintz, N. (2013). GENSAT BAC cre-recombinase driver lines to study the functional organization of cerebral cortical and basal ganglia circuits. Neuron *80*, 1368–1383.

Hartman, K.N., Pal, S.K., Burrone, J., and Murthy, V.N. (2006). Activity-dependent regulation of inhibitory synaptic transmission in hippocampal neurons. Nat. Neurosci. *9*, 642–649.

Harvey, C.D., Yasuda, R., Zhong, H., and Svoboda, K. (2008). The spread of Ras activity triggered by activation of a single dendritic spine. Science *321*, 136–140.

Hengen, K.B., Lambo, M.E., Van Hooser, S.D., Katz, D.B., and Turrigiano, G.G. (2013). Firing rate homeostasis in visual cortex of freely behaving rodents. Neuron *80*, 335–342.

Hengen, K.B., Torrado Pacheco, A., McGregor, J.N., Van Hooser, S.D., and Turrigiano, G.G. (2016). Neuronal firing rate homeostasis is inhibited by sleep and promoted by wake. Cell *165*, 180–191.

Holtmaat, A., Bonhoeffer, T., Chow, D.K., Chuckowree, J., De Paola, V., Hofer, S.B., Hübener, M., Keck, T., Knott, G., Lee, W.-C.A., et al. (2009). Long-term, high-resolution imaging in the mouse neocortex through a chronic cranial window. Nat. Protoc. *4*, 1128–1144.

Iacaruso, M.F., Gasler, I.T., and Hofer, S.B. (2017). Synaptic organization of visual space in primary visual cortex. Nature *547*, 449–452.

Ibata, K., Sun, Q., and Turrigiano, G.G. (2008). Rapid synaptic scaling induced by changes in postsynaptic firing. Neuron *57*, 819–826.

Ibrahim, L.A., Mesik, L., Ji, X.-Y., Fang, Q., Li, H.-F., Li, Y.-T., Zingg, B., Zhang, L.I., and Tao, H.W. (2016). Cross-modality sharpening of visual cortical processing through layer-1-mediated inhibition and disinhibition. Neuron *89*, 1031–1045.

Iurilli, G., Ghezzi, D., Olcese, U., Lassi, G., Nazzaro, C., Tonini, R., Tucci, V., Benfenati, F., and Medini, P. (2012). Sound-driven synaptic inhibition in primary visual cortex. Neuron *73*, 814–828.

Ju, W., Morishita, W., Tsui, J., Gaietta, G., Deerinck, T.J., Adams, S.R., Garner, C.C., Tsien, R.Y., Ellisman, M.H., and Malenka, R.C. (2004). Activity-dependent regulation of dendritic synthesis and trafficking of AMPA receptors. Nat. Neurosci. *7*, 244–253.

Kaneko, M., Stellwagen, D., Malenka, R.C., and Stryker, M.P. (2008). Tumor necrosis factor-alpha mediates one component of competitive, experience-dependent plasticity in developing visual cortex. Neuron *58*, 673–680.

Keck, T., Scheuss, V., Jacobsen, R.I., Wierenga, C.J., Eysel, U.T., Bonhoeffer, T., and Hübener, M. (2011). Loss of sensory input causes rapid structural changes of inhibitory neurons in adult mouse visual cortex. Neuron *71*, 869–882.

Keck, T., Keller, G.B., Jacobsen, R.I., Eysel, U.T., Bonhoeffer, T., and Hübener, M. (2013). Synaptic scaling and homeostatic plasticity in the mouse visual cortex in vivo. Neuron *80*, 327–334.

Keck, T., Hübener, M., and Bonhoeffer, T. (2017). Interactions between synaptic homeostatic mechanisms: an attempt to reconcile BCM theory, synaptic scaling, and changing excitation/inhibition balance. Curr. Opin. Neurobiol. *43*, 87–93.

Keller, G.B., Bonhoeffer, T., and Hübener, M. (2012). Sensorimotor mismatch signals in primary visual cortex of the behaving mouse. Neuron *74*, 809–815.

Kim, J., Tsien, R.W., and Alger, B.E. (2012). An improved test for detecting multiplicative homeostatic synaptic scaling. PLoS ONE *7*, e37364.

Lee, S.-H., Kwan, A.C., Zhang, S., Phoumthipphavong, V., Flannery, J.G., Masmanidis, S.C., Taniguchi, H., Huang, Z.J., Zhang, F., Boyden, E.S., et al. (2012). Activation of specific interneurons improves V1 feature selectivity and visual perception. Nature *488*, 379–383.

Lewitus, G.M., Pribiag, H., Duseja, R., St-Hilaire, M., and Stellwagen, D. (2014). An adaptive role of TNFα in the regulation of striatal synapses. J. Neurosci. *34*, 6146–6155.

Lewitus, G.M., Konefal, S.C., Greenhalgh, A.D., Pribiag, H., Augereau, K., and Stellwagen, D. (2016). Microglial TNF-α suppresses cocaine-induced plasticity and behavioral sensitization. Neuron *90*, 483–491.

Lin, S.-C., and Bergles, D.E. (2004). Synaptic signaling between neurons and glia. Glia *47*, 290–298.

Loewenstein, Y., Kuras, A., and Rumpel, S. (2011). Multiplicative dynamics underlie the emergence of the log-normal distribution of spine sizes in the neocortex in vivo. J. Neurosci. *31*, 9481–9488.

López-Bendito, G., Sturgess, K., Erdélyi, F., Szabó, G., Molnár, Z., and Paulsen, O. (2004). Preferential origin and layer destination of GAD65-GFP cortical interneurons. Cereb. Cortex *14*, 1122–1133.

Losonczy, A., Makara, J.K., and Magee, J.C. (2008). Compartmentalized dendritic plasticity and input feature storage in neurons. Nature *452*, 436–441.

Makara, J.K., Losonczy, A., Wen, Q., and Magee, J.C. (2009). Experience-dependent compartmentalized dendritic plasticity in rat hippocampal CA1 pyramidal neurons. Nat. Neurosci. *12*, 1485–1487.

Makino, H., and Malinow, R. (2011). Compartmentalized versus global synaptic plasticity on dendrites controlled by experience. Neuron *72*, 1001–1011.

Mao, R., Schummers, J., Knoblich, U., Lacey, C.J., Van Wart, A., Cobos, I., Kim, C., Huguenard, J.R., Rubenstein, J.L.R., and Sur, M. (2012). Influence of a subtype of inhibitory interneuron on stimulus-specific responses in visual cortex. Cereb. Cortex *22*, 493–508.

Niell, C.M., and Stryker, M.P. (2010). Modulation of visual responses by behavioral state in mouse visual cortex. Neuron *65*, 472–479.

Noguchi, J., Nagaoka, A., Watanabe, S., Ellis-Davies, G.C.R., Kitamura, K., Kano, M., Matsuzaki, M., and Kasai, H. (2011). In vivo two-photon uncaging of glutamate revealing the structure-function relationships of dendritic spines in the neocortex of adult mice. J. Physiol. *589*, 2447–2457.

Oh, W.C., Parajuli, L.K., and Zito, K. (2015). Heterosynaptic structural plasticity on local dendritic segments of hippocampal CA1 neurons. Cell Rep. *10*, 162–169.

Pakan, J.M., Lowe, S.C., Dylda, E., Keemink, S.W., Currie, S.P., Coutts, C.A., and Rochefort, N.L. (2016). Behavioral-state modulation of inhibition is context-dependent and cell type specific in mouse visual cortex. eLife *5*, e14985.

Poirazi, P., Brannon, T., and Mel, B.W. (2003). Pyramidal neuron as two-layer neural network. Neuron *37*, 989–999.

Roth, M.M., Dahmen, J.C., Muir, D.R., Imhof, F., Martini, F.J., and Hofer, S.B. (2016). Thalamic nuclei convey diverse contextual information to layer 1 of visual cortex. Nat. Neurosci. *19*, 299–307.

Rutherford, L.C., Nelson, S.B., and Turrigiano, G.G. (1998). BDNF has opposite effects on the quantal amplitude of pyramidal neuron and interneuron excitatory synapses. Neuron *21*, 521–530.

Saleem, A.B., Ayaz, A., Jeffery, K.J., Harris, K.D., and Carandini, M. (2013). Integration of visual motion and locomotion in mouse visual cortex. Nat. Neurosci. *16*, 1864–1869.

Steinmetz, C.C., and Turrigiano, G.G. (2010). Tumor necrosis factor-α signaling maintains the ability of cortical synapses to express synaptic scaling. J. Neurosci. *30*, 14685–14690.

Stellwagen, D., and Malenka, R.C. (2006). Synaptic scaling mediated by glial TNF-alpha. Nature *440*, 1054–1059.

Sutton, M.A., Ito, H.T., Cressy, P., Kempf, C., Woo, J.C., and Schuman, E.M. (2006). Miniature neurotransmission stabilizes synaptic function via tonic suppression of local dendritic protein synthesis. Cell *125*, 785–799.

Takahashi, N., Kitamura, K., Matsuo, N., Mayford, M., Kano, M., Matsuki, N., and Ikegaya, Y. (2012). Locally synchronized synaptic inputs. Science *335*, 353–356.

Tohmi, M., Meguro, R., Tsukano, H., Hishida, R., and Shibuki, K. (2014). The extrageniculate visual pathway generates distinct response properties in the higher visual areas of mice. Curr. Biol. *24*, 587–597.

Tremblay, R., Lee, S., and Rudy, B. (2016). GABAergic interneurons in the neocortex: from cellular properties to circuits. Neuron *91*, 260–292.

Turrigiano, G. (2011). Too many cooks? Intrinsic and synaptic homeostatic mechanisms in cortical circuit refinement. Annu. Rev. Neurosci. *34*, 89–103.

Turrigiano, G. (2012). Homeostatic synaptic plasticity: local and global mechanisms for stabilizing neuronal function. Cold Spring Harb. Perspect. Biol. *4*, a005736.

Turrigiano, G.G., Leslie, K.R., Desai, N.S., Rutherford, L.C., and Nelson, S.B. (1998). Activity-dependent scaling of quantal amplitude in neocortical neurons. Nature *391*, 892–896.

Wallace, W., and Bear, M.F. (2004). A morphological correlate of synaptic scaling in visual cortex. J. Neurosci. *24*, 6928–6938.

Wilson, D.E., Whitney, D.E., Scholl, B., and Fitzpatrick, D. (2016). Orientation selectivity and the functional clustering of synaptic inputs in primary visual cortex. Nat. Neurosci. *19*, 1003–1009.

Winnubst, J., Cheyne, J.E., Niculescu, D., and Lohmann, C. (2015). Spontaneous activity drives local synaptic plasticity in vivo. Neuron *87*, 399–410.

Xu, N.L., Harnett, M.T., Williams, S.R., Huber, D., O'Connor, D.H., Svoboda, K., and Magee, J.C. (2012). Nonlinear dendritic integration of sensory and motor input during an active sensing task. Nature *492*, 247–251.

Yoshitake, K., Tsukano, H., Tohmi, M., Komagata, S., Hishida, R., Yagi, T., and Shibuki, K. (2013). Visual map shifts based on whisker-guided cues in the young mouse visual cortex. Cell Rep. *5*, 1365–1374.

Yu, L.M.Y., and Goda, Y. (2009). Dendritic signalling and homeostatic adaptation. Curr. Opin. Neurobiol. *19*, 327–335.

Zmarz, P., and Keller, G.B. (2016). Mismatch receptive fields in mouse visual cortex. Neuron *92*, 766–772.

STAR★METHODS

KEY RESOURCES TABLE

REAGENT or RESOURCE	SOURCE	IDENTIFIER
Antibodies		
Chicken Anti-GFP	Abcam	RRID: AB_300798
anti-GluA2 mouse	NeuroMab	RRID:AB_2232661
anti-GRIP1 rabbit	Abcam	RRID:AB_880303
goat anti-chicken Alexa Fluor 488	Invitrogen	RRID:AB_142924
goat anti-mouse IgG1 Alexa Fluor 568	Invitrogen	RRID:AB_141611
goat anti-rabbit Alexa Fluor 647	Invitrogen	RRID:AB_141663
Bacterial and Virus Strains		
AAV2/1-*ef1α*-DiO-GCaMP6f	FMI Vector Core	N/A
AAV2/1-*ef1α*-GCaMP6f	FMI Vector Core	N/A
Chemicals, Peptides, and Recombinant Proteins		
XPro1595	Xencor, Inc.	N/A
Isoflurane (Attane)	Provet	CAS 26221-73-3
Dental Cement (Paladur)	Heraeus Kulzer	CAS 9066-86-8
Ketamine	Pfizer	CAS 1867-66-9
Xylazine	Rompun	CAS 7361-61-7
Emla Cream 5%	AstraZeneca	CAS 137-58-6, CAS 721-50-6
Tetrodotoxin	Tocris	CAS 4368-28-9
D Glucose	Sigma-Aldrich	CAS 50-99-7
NaCl,	Tocris	CAS 7647-14-5
KCl	Tocris	CAS 7447-40-7
$NaHCO_3$	Tocris	CAS 144-55-8
NaH_2PO_4	Tocris	CAS 7558-80-7
$CaCl_2$	Sigma-Aldrich	CAS 10043-52-4
$MgSO_4$	Sigma-Aldrich	CAS 7487-88-9
Bovine Serum Albumin	Sigma-Aldrich	CAS 9048-46-8
Triton X	Sigma-Aldrich	CAS 9002-93-1
Paraformaldehyde	Sigma-Aldrich	CAS 30525-89-4
Experimental Models: Organisms/Strains		
Mouse: Thy-1 GFP-M line	JAX	RRID:IMSR_JAX:007788
Mouse: GAD-65-GFP line	(López-Bendito et al., 2004)	N/A
Mouse: Tg(Npy-cre)RH26Gsat	MMRRC	MMRRC_034810-UCD
Mouse: C57BL6/J	Charles River Laboratories	N/A
Software and Algorithms		
MATLAB	The MathWorks, Inc.	RRID: SCR_001622
LabView	National Instruments	RRID: SCR_014325
Sigmaplot13	Systat Software, Inc.	N/A
MiniAnalysis Programme	Synaptosoft, Inc.	N/A
LAS-X software	Leica	N/A
Scanimage	Vidrio technolgies	RRID: SCR_014307
Ephus	Vidrio technolgies	N/A
ImageJ	NIH	RRID: SCR_003070

CONTACT FOR REAGENT AND RESOURCE SHARING

Further information and requests for resources and reagents should be directed to and will be fulfilled by the Lead Contact, Tara Keck (t.keck@ucl.ac.uk).

EXPERIMENTAL MODEL AND SUBJECT DETAILS

Animals

Experiments were conducted according to the United Kingdom Animals (Scientific Procedures) Act 1986 or were approved by the Veterinary Department of the Canton of Basel-Stadt, Switzerland. We used adult male and female mice (P60-120; n = 60 *Thy1*-GFP; n = 59 *GAD65*-GFP; n = 13 C57BL/6; n = 14 Tg(Npy-cre)RH26Gsat). All animals were sex and age matched within experimental groups. Mice were housed with littermates (2-6 mice depending on litter size) and kept on a 12 hr light-dark cycle. Imaging experiments were time matched during the light cycle. C57BL/6 mice were used for functional imaging experiments of excitatory neurons. As a majority (~90%) of inhibitory neurons with dendritic spines in the mouse visual cortex in the *GAD65*-GFP line express NPY (Keck et al., 2011), we used Tg(Npy-cre)RH26Gsat mice (Gerfen et al., 2013) for functional imaging of inhibitory neurons. Mice expressing enhanced green fluorescent protein (GFP) under the *Thy1* promoter, GFP-M line (Feng et al., 2000) were used for excitatory cell structural imaging, electrophysiology and immunohistochemistry experiments. Mice expressing GFP under the *GAD65* promoter (López-Bendito et al., 2004) were used for inhibitory cell structural imaging, electrophysiology and immunohistochemistry experiments, where inhibitory neurons with dendritic spines were used in all experiments.

METHOD DETAILS

Surgery

For in vivo imaging experiments, cranial windows were surgically implanted over the right hemisphere of monocular visual cortex, as described previously (Holtmaat et al., 2009). We made a craniotomy in ketamine/xylazine (0.15 mg/g and 0.015 mg/g of body weight respectively) anesthetized mice and replaced the skull with a glass coverslip that was attached to the bone with dental cement. For functional imaging experiments, mice were injected with AAV2/1-*ef1α*-GCaMP6f (C57BL/6 mice) or AAV2/1-*ef1α*-DiO-GCaMP6f (Tg(Npy-cre)RH26Gsat mice) before the glass coverslip was positioned. Mice were allowed to recover for at least 28 days after surgery before imaging commenced. For monocular enucleation, we applied lidocaine (Emla cream) to the area around the left eye in anesthetized mice prior to surgical removal of the eye. Control 'sham-enucleated' mice were given time-matched anesthesia. Animals were randomly assigned into enucleation or sham-enucleation groups, such that half of each litter was in each group. We used intrinsic signal imaging before enucleation to localize the monocular visual cortex as described previously (Keck et al., 2013). For functional imaging using GCaMP6f, brief isoflurane sedation (approximately 10 s) was used to head-fix the mice before imaging either 4 hr (excitatory cells) or 24 hr (inhibitory cells) after enucleation or sham-enucleated controls. For structural imaging, animals were imaged while anaesthetised with ketamine/xylazine either 1) every 24 hr, twice before enucleation or sham-enucleation and twice after (Figure 1C) or 2) once before, then every 4 hr for 8 hr total after enucleation (or sham-enucleated controls) where the animals remained anaesthetized throughout (Figure 3E). For the in vivo imaging and slice electrophysiology experiments with the TNF-α inhibitor, mice were injected with XPro1595 (Lewitus et al., 2014) (Xencor, Inc) at a dose of 10 mg/kg twice before enucleation and once after (-72,-24 and 24 hr).

Immunohistochemistry

We transcardially perfused either deprived (48 hr post-enucleation) or anesthesia matched sham-enucleated control *GAD65*-GFP or *Thy1*-GFP mice with phosphate-buffered saline (PBS), then 4% paraformaldehyde (PFA) and performed immunohistochemistry against GFP, GluA2 and GRIP1. Coronal brain slices were prepared from primary visual cortex at a thickness of 60 μm and incubated in blocking agent (3% Bovine Serum Albumin, 0.25% Triton-X in PBS) and then in primary antibody for 20 hr at room temperature. Slices were then washed 3 times for 10 min in PBS and incubated with secondary antibody for 3 hr. The slices were again washed 3 times for 10 min in PBS before mounting them on a coverslip. We used the following antibodies: anti-GFP chicken polyclonal (Abcam, ab13970, 1:1000), anti-GluA2 mouse monoclonal (NeuroMab, 75-002, 1:300), anti-GRIP1 rabbit polyclonal (Abcam, ab25963, 1:100), goat anti-chicken Alexa Fluor 488 (Invitrogen, A11039, 1:500), goat anti-mouse IgG1 Alexa Fluor 568 (Invitrogen, A21124, 1:500), goat anti-rabbit Alexa Fluor 647 (Invitrogen, A21244, 1:500). Cells located in the monocular portion of primary visual cortex, as identified by stereotaxic coordinates, were imaged using a Leica SP8 confocal microscope (LAS-X software). Images were collected with a Leica 63 × 1.40 NA oil CS2 objective (15506350) and a zoom of 4 ×. Parameters of the collected images were 1024 pixels × 1024 pixels, 46 μm × 46 μm, 0.5 μm z-step. Custom written software was developed in MATLAB and used to analyze GFP, GluA2 and GRIP1 fluorescence intensity profiles, which were measured in ImageJ.

Electrophysiology

Targeted whole-cell patch clamp electrophysiological recordings of mEPSCs from layer 2/3 *GAD65*-GFP or layer 5 *Thy1*-GFP positive neurons were made under epi-fluorescence illumination on a custom built set-up as described previously (Barnes et al., 2015;

Keck et al., 2013). In brief, we prepared acute slices of visual cortex from mice 48 hr after monocular enucleation (or anesthesia matched controls) and recorded in the monocular visual cortex contralateral to the deprived eye. We measured mEPSC recordings in the presence of 1 μM TTX at room temperature, in recording ACSF (in mM, 126 NaCl, 3.5 KCl, 25 $NaHCO_3$, 1 NaH_2PO_4, 25 D-glucose, 2 $CaCl_2$ and 1 $MgSO_4$ saturated with 95% O_2 / 5% CO_2). Recordings were disregarded if the cellular resistance was lower than 200 MΩ, the resting membrane potential was more positive than -60mV, or the cellular resistance or resting membrane potential changed by more than 10% of initial values throughout the duration of the experiment.

Model

We developed a model neuron consisting of *Nbr* = 20 branches, each of which has *Nsyn* = 15 synapses. The input patterns x_{ij} were generated from a random uniform distribution from 0.5 to 1.5 (mean of 1), where *i* is the branch index (from 1 to *Nbr*) and $_j$ is the synaptic index (from 1 to *Nsyn*). Each dendritic branch computes the weighted (W_{ij}) sum of its activated inputs, which is then passed through a non-linearity (a sigmoid function) to mimic the nonlinear summation of dendrites (based on Poirazi et al., 2003). The output of the neuron *y* is calculated by summing the branch activations and then passing the result through an additional non-linearity, as described by:

$$y = f^n\left(\frac{1}{Nbr}\sum_i w_i^b f^b\left(\frac{1}{Nsyn}\sum_j w_{ij}x_{ij}\right)\right),$$

where the nonlinearity *f* is a sigmoid, $f(x) = 1/1 + e^{-\beta(x-\mu)}$, where $\beta = 0.7$, $\mu = 33$ are constants for the branch non-linearity f^b, $\beta = 5$, $\mu = 0.5$ are constants for the neuron non-linearity f^n, and w_i^b is the weight of the branch (Poirazi et al., 2003).

The weights w_{ij} are taken from a lognormal distribution with a mean of 30 [a.u.] spanning from 20-150 [a.u.], based on observations from our experimental data. Conditions of deprived animals are simulated so that 50% of the dendritic branches undergo spine loss, in which on average 10% of randomly selected weights are set to zero. We then apply two possible normalizations to mimic synaptic scaling: either the weights are normalized per branch (branch specific) so that all branches have the same total synaptic weight, or across the whole neuron (global). Increases in input weight and total input loss are balanced so that the total synaptic weight of the neuron is the same in both conditions. Note that the weights that are removed during the deprivation are kept at zero. The neuron was presented with 1000 random input patterns, where a subset of the inputs on a branch is active at one given time. The mutual information between the output *y* and the inputs x_{ij} (discretized in 10 bins) was computed for the same input patterns in the model conditions of global and branch specific scaling. The process was repeated 100 times to estimate the mean and the standard deviation.

QUANTIFICATION AND STATISTICAL ANALYSIS

Functional imaging and analysis

Measurements and analysis of functional imaging data were conducted as described previously (Barnes et al., 2015; Keck et al., 2013; Keller et al., 2012). Functional calcium imaging of volumes of cortex was performed on a custom built two-photon microscope with an 8 kHz resonance scanner (Cambridge Technology) and a high power objective Z-piezo stage (Physik Instrumente), using a MaiTai eHP laser with a DeepSee prechirp unit (Newport/Spectra Physics) or a Chameleon Vision S (Coherent) set to 910 nm and a Nikon 16 × 0.8 NA objective, as described previously (Barnes et al., 2015). Data were acquired with a 250 MHz digitizer (National Instruments) and pre-processed with a custom programmed field programmable gate array (FPGA) (National Instruments). The dynamic range of both the amplifier and the PMT exceeded the digitization range and the data acquisition software automatically detected digital saturation of all pixels. Animals were allowed to habituate to the setup, while head-fixed and running freely on a spherical treadmill (Keller et al., 2012). Animals were presented with vertical visual gratings whose movement was coupled to the animal's movement, alternating with periods of darkness for three minutes each, repeated twice. Imaging data were full-frame registered using a custom written registration algorithm. To remove slow signal changes in raw fluorescence traces, the 8th-percentile value of the fluorescence distribution in a ± 15 s window was subtracted from the raw fluorescence signal (Dombeck et al., 2007). Dendritic branchpoints of layer 5 pyramidal cells were identified in the upper layers, where they could be traced below layer 4 to the cell body. Dendritic branchpoints in NPY positive cells were identified and traced to the cell body in layer 2/3. To examine the variation in activity between two dendritic branches at a branchpoint, we measured the correlation between the signals measured in the two dendritic branches. To determine the expected variation within a dendritic branch given our experimental protocol, we measured the correlation between signals in two regions within the same dendritic branch that were 10 μm apart. Finally, we measured the correlation with a dendrite that was in the same imaging region, but on a different cell. For all three of the above descriptions, we also measured the mutual information between the two signals and took the integral of the $\Delta F/F_0$ signal to measure the total activity. Between the two branches and within a branch, 'branch 1' was randomly chosen, but was consistent across all conditions. To identify calcium transients that were specific to individual branches (branch specific events), we based our criteria on previously published work (Cichon and Gan, 2015). Calcium transients that occurred in one branch and not the other had to have a peak response of at least 15% $\Delta F/F_0$. We measured the frequency and peak amplitude of all identified branch specific calcium events in individual branches. We then measured the total integral of branch specific event activity and all activity in each branch, and measured the percentage of all activity that is attributable to the branch specific events for each dendrite. Finally, we subtracted

the total integral of branch specific calcium events in branch 1 of the pair from branch 2 of the pair and took the absolute value. This gave us a measure of how different the overall activity between the branches was in total due to dendritic branch specific calcium events. We made these measurements for both enucleated animals and sham-enucleated controls.

Structural imaging and analysis

In vivo structural imaging and analysis was performed as described previously (Barnes et al., 2015; Keck et al., 2013). Briefly, we used a two-photon microscope with a MaiTai BB laser with a DeepSee prechirp unit (Newport/Spectra Physics) set to 909 nm and an Olympus 40 × 0.8 NA water immersion objective. The average laser power was kept below 50 mW. For image acquisition, we used Scanimage freeware (VidrioTech). Image parameters were: 64 × 64 μm, 512 × 512 pixels, 0.5 μm step in depth. Three-dimensional in vivo images of dendritic spines were analyzed in ImageJ (NIH), blind to experimental condition and to time. All further analyses were done in custom written MATLAB software (Mathworks, Inc.). Density was calculated as all spines that were clearly visible per micrometer of dendrite. Spine size was calculated as integrated brightness, as described previously (Barnes et al., 2015; Keck et al., 2013). Briefly, spine intensity was background subtracted and normalized by the intensity of the adjacent dendrite to account for differences in image intensity between imaging time points. Only spines visible in the x-y plane were measured. Because spine size is normalized to the parent dendrite, changes in the width of the dendrite could potentially affect our spine size measurements. We measured dendrite width over our imaging time course at the same position before and after enucleation, with the analyzer blind to time. We next determined the degree of spine size measurement noise attributable to changes in dendrite width by normalizing our spine size measurements at time point 0 hr (just prior to deprivation) to the dendrite at time point 48 hr after deprivation. We compared this value to the same 0 hr spine size normalized to the dendrite at 0 hr. This measure gives us an idea of the variation in spine size that would result from any changes in the dendrite width (Figures S3J and S3L–S3N).

Dendritic branches imaged in vivo were defined as undergoing increases in spine size (“increasing branches”) if they had a significant increase of at least 10% (or 1.1 when normalized) in average normalized spine size (> 90th percentile of control distribution), when normalized to baseline for individual spines. Our results did not change qualitatively when using different thresholds (Figures S2C, S2D, and S3F). Branches also needed to have more than 45% (> 90th percentile of control distribution) of dendritic spines increasing in size above the 1.1 value. This constraint prevents single large outlier spines from determining the classification of a dendrite as increasing. The same criterion was applied to dendritic branches from immunohistochemistry experiments (which are from a single time point), but here individual spines in deprived animals were normalized to the average spine size in control animals. To determine the chance levels of dendritic branches imaged in vivo undergoing increases in spine size given the total number of individual dendritic spines that increase in size, we generated 100 datasets of 30 simulated dendritic branches, in which we randomly chose 25 dendritic spines, with replacement, from all dendritic spines measured in deprived animals. We then calculated the average spine size and proportion of spines increasing in size for these simulated dendritic branches to determine the fraction of branches that met our criteria for a branch undergoing increases in spine size by chance. For analyses where we grouped branches into increasing, same and decreasing, increasing branches used the same criteria as above. Of the remaining branches, same branches had an average change in spine size between 0.9 and 1.1 and branches that decrease had an average change in spine size below 0.9. Related to the timing of spine size increase and spine loss, we examined the time point at which the spine size increased according to our criteria and determined the fraction of branches that had a decrease in spine size in the previous time point. (Inhibitory: Increasing branches = 81%, Non-increasing branches = 27%, p = 0.007, z-test. Excitatory: Increasing branches = 80%, Non-increasing branches = 30%, p = 0.025, z-test.).

For the cluster analysis, we measured whether a spine increased or not for every spine on a dendrite based on a scaling threshold (1.1, 1.15, 1.20), measured as the size at 48 hr after enucleation and normalized to baseline for each individual spine. For all increasing spines Sp_0, we calculated the proportion of all neighboring spines that also increased by the same threshold a given distance away from the increasing spine, either in 4 μm bins, based on the average interspine interval (inhibitory neurons: 4.2 ± 0.1 μm; excitatory neurons: 3.5 ± 0.4 μm), or 10 μm bins. Positions of dendritic spines on branches with increasing spines were then randomly shuffled, so that branches had the same number of spines as before with the same distribution of distances, but not in the same spatial order (Figure 2A). The same calculation for clustering was then performed on the shuffled data. The population distribution (Figures 2B and 2C, black) was calculated in the same way as above, but for all spines in the population serving as Sp_0 (increasing and not), rather than just spines that increase in size. Finally, the same calculations were performed specifically for spines, Sp_0, that did not increase in size (‘same’, normalized size between 0.9 and 1.1) or decreased in size (normalized size below 0.9), with neighboring spines that did exceed the scaling threshold. Based on Fu et al., 2012, spine position was measured from the position where the spine touched the dendritic shaft, without consideration for the direction in which the spine extended. Thus, spines that extend directly opposite one another on the dendrite were considered 0 μm apart. Distance between spines was calculated as the distance along the dendrite between the positions where each respective spine meets the dendrite. We ran the clustering analysis for all spines imaged, so not all spines had the same number of neighbors in each distance bin. The average number of stable spines per dendritic branch was inhibitory: 13 ± 1 stable spines; excitatory: 16 ± 2 stable spines. The different branch (DB) condition was calculated for each spine by randomly choosing a single spine on a different branch of the same cell. To get a DB probability for each spine Sp_0, we repeated this sampling, with replacement, ten times. We also calculated the interspine distance between lost spines to determine if there was clustering of spine loss. We measured all lost spines’ positions, independent of the time point in which they were lost. We then measured the distance between each lost spine and its nearest lost spine neighbor. We did this analysis for both control and deprived

animals. Then, on dendrites from deprived animals, we shuffled the position of the all spines on the dendrite (lost, stable and new), and again calculated the interspine distance between lost spines.

Immunohistochemistry image analysis

The peak of the GFP intensity profile was used to localize the values from the intensity profiles of both the GluA2 and GRIP1 images within individual spines. To control for non-specific immunostaining, the GluA2 and GRIP1 intensity values were then normalized to a 90 degree rotation of the original fluorescence image (background value) within the spine. We measured the peak intensity of GluA2 that co-localized with GRIP1. Co-localization was determined as dendritic spines that had an intensity of both GRIP1 and GluA2 that was greater than two standard deviations above their respective background fluorescence. Measurements were then averaged for all spines on the entire dendrite that co-localized with GRIP1. For a given spine, the GluA2 to spine size ratio values were calculated by dividing the normalized GluA2 intensity value at a spine by the integrated brightness of that spine **(**Figure S1Z**)**. We measured spine size in these slices in the same way described for in the in vivo spine size measurements. Branch order measurements were made in all slices where we could trace the analyzed branches back to the soma. All data collection and analysis was done blind to experimental condition.

Electrophysiology analysis

Analysis of mEPSCs was conducted blind to experimental condition using Mini Analysis (Synaptosoft, Inc.). Parameters were as described previously, with 30-50 mEPSC events taken per cell (Barnes et al., 2015; Keck et al., 2013). Briefly, amplitudes were greater than 5 pA and 20%–80% rise times of less than 1 ms. In order to determine whether the mEPSC amplitude distribution exhibited multiplicative scaling we adapted a previously published approach (Kim et al., 2012). A range of multiplicative scaling factors were used to scale either 100% or 50% of the control mEPSC amplitude distribution to the deprived distribution. We then compared each (50% or 100%) scaled control distribution to the measured deprived distribution using Kolomogrov-Smirnov tests (K-S test) for each scaling factor tested (Figures S2I–S2P). The highest non-significant p value from the K-S test was considered the best scaling factor. For cases where 50% of the control distribution were scaled, we randomly sampled values based on the underlying probability distribution of the control sample, which was non-parametric. We then combined these ‘scaled’ values with the remaining non-scaled values to get a 50% scaled distribution.

Statistics

Statistical analyses were performed either in MATLAB or SigmaPlot. Data were tested for equal variance and normality (Shapiro-Wilk test) and then comparisons were made using parametric or non-parametric tests, as appropriate (t test, paired t test, z-test Chi-square test, Wilcoxon Signed Rank test, Mann Whitney Rank Sum test, One-Way ANOVA with Holm-Sidak post hoc test, repeated-measures ANOVA with Holm-Sidak post hoc test, ANOVA on Ranks with Dunn’s Method post hoc test or a Two-Way ANOVA with Holm-Sidak post hoc test, Kullback-Leibler divergence test). For normalized dendritic spine size, data were $\log_{10}$ transformed (Loewenstein et al., 2011) before statistical tests were run, as noted in the text. Statistical tests were two-sided. Correlation coefficients were calculated with a Spearman’s rank or Pearson’s correlation coefficient. A power analysis was performed to ensure we used a sufficient sample size. Specific statistical tests used for all figures along with the number of samples and details of center and dispersion measures can be found in supplemental tables S1-S3 and the figure legends.

DATA AND SOFTWARE AVAILABILITY

Requests for data and software should be directed to the Lead Contact, Tara Keck (t.keck@ucl.ac.uk) and will be made available upon reasonable request.

Code availability

The code used for image registration and data acquisition of the functional data is available at https://sourceforge.net/projects/iris-scanning/.

Neuron, Volume *96*

Supplemental Information

Deprivation-Induced Homeostatic Spine Scaling *In Vivo* Is Localized to Dendritic Branches that Have Undergone Recent Spine Loss

Samuel J. Barnes, Eleonora Franzoni, R. Irene Jacobsen, Ferenc Erdelyi, Gabor Szabo, Claudia Clopath, Georg B. Keller, and Tara Keck

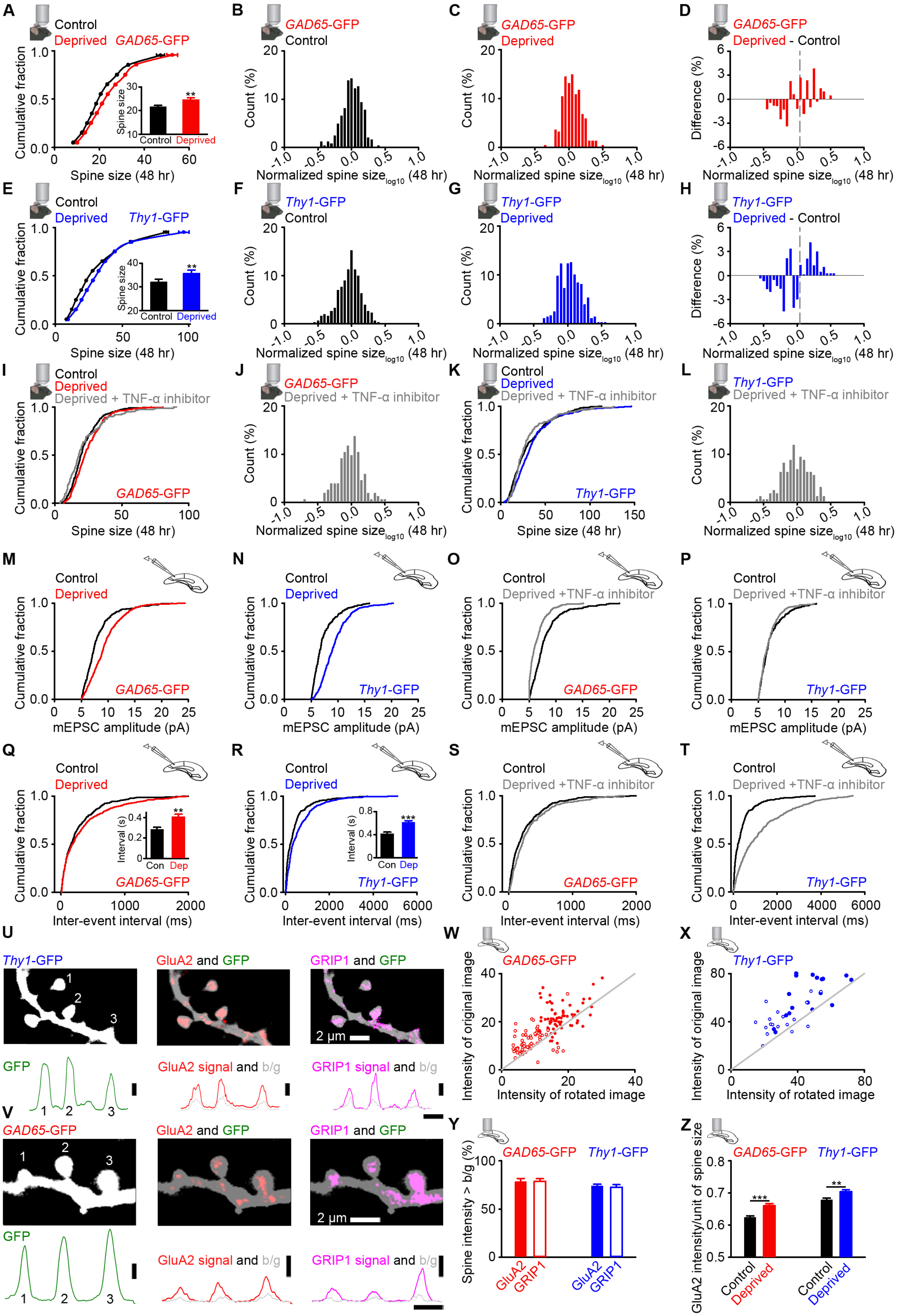

Supplementary Figure 1

Figure S1, Related to Figure 1.

A,E,I,K) Cumulative distribution of raw spine size values for inhibitory (A,I) and excitatory (E,K) neurons *in vivo* 48 hours after deprivation in control mice (black), deprived mice (red/blue) and deprived mice injected with the TNF-α inhibitor (I,K,gray). (Statistics on distributions of all spines. I, Control versus deprived, p=0.011; Deprived versus deprived+TNF-α inhibitor, p<0.001; Control versus deprived+TNF-α inhibitor, p=0.665; One-Way ANOVA on Ranks with post-hoc test. K, Control versus deprived, p=0.021; Deprived versus deprived+TNF-α inhibitor, p=0.013; Control versus deprived+TNF-α inhibitor, p=0.998; One-Way ANOVA on Ranks with post-hoc test). Insets in A and E show average spine size (Control versus deprived: A, p=0.002; E, p=0.006, t-test on log-transformed data).

B,C,F,G,J,L) Distribution of normalized (48 hours normalized to baseline) spine size values following a log-transform for inhibitory (B,C,J) and excitatory (F,G,L) neurons *in vivo* 48 hours after (sham) enucleation in control mice (B,F, black), deprived mice (C,G, red/blue) and deprived mice injected with the TNF-α inhibitor (J,L, gray). (Statistics on distribution of all spines. B,C,J, Control versus deprived, p=0.008; Control versus Deprived+TNF-α inhibitor, p=0.064; Two-Way ANOVA with post-hoc test on log-transformed data. F,G,L, Control versus deprived, p<0.001; Control versus deprived+TNF-α inhibitor, p=0.923; Two-Way ANOVA with post-hoc test on log-transformed data – Note that these are the same statistics as in Table S1 for Fig. 1D,E with the Two-Way ANOVA).

D,H) Distribution of the difference in control and deprived (48 hours) log-transformed spine size distributions between for inhibitory (D) and excitatory (H) neurons. Gray dashed line shows 1.1 threshold for spine size increase.

M,N,O,P) Cumulative distribution of mEPSC amplitude for inhibitory (M,O) and excitatory (N,P) neurons in slices prepared 48 hours after (sham) deprivation from control mice (M-P black), deprived mice (M-N, red/blue) and deprived mice injected with the TNF-α inhibitor (O-P, gray). (Statistics on distribution of all events. M,O, Control versus deprived, p<0.001; Control versus deprived+TNF-α inhibitor, p<0.001, One-Way ANOVA on Ranks with post-hoc test. N,P, Control versus deprived, p<0.001; Control versus deprived+TNF-α inhibitor, p=0.456, One-Way ANOVA on Ranks with post-hoc test). For panels M,O, control n=9 cells, deprived n=16 cells,

deprived+TNF-α inhibitor n=10 cells. For panels N,P, control n=10 cells, deprived n=15 cells, deprived+TNF-α inhibitor n=13 cells.

Q,R,S,T) Distribution of mEPSC inter-event intervals for inhibitory (Q,S) and excitatory (R,T) neurons in slices prepared 48 hours after (sham) deprivation from control mice (Q-T black), deprived mice (Q-R, red/blue) and deprived mice injected with the TNF-α inhibitor (S-T, gray). (Statistics on distributions of all events. Q,S, Control versus deprived, $p=0.007$; Control versus deprived+TNF-α inhibitor, $p=0.044$, One-Way ANOVA on Ranks with post-hoc test. R,T, Control versus deprived, $p<0.001$; Control versus deprived+TNF-α inhibitor, $p<0.001$, One-Way ANOVA on Ranks with post-hoc test). Insets, inter-event interval average (Control versus deprived: Q, $p=0.007$; R, $p<0.001$, t-test on log-transformed data). For panels Q,S, control n=9 cells, deprived n=16 cells, deprived+TNF-α inhibitor n=10 cells. For panels R,T, control n=10 cells, deprived n=15 cells, deprived+TNF-α inhibitor n=13 cells.

U,V) Top, example images of a dendritic section from either an excitatory (U) or inhibitory (V) neuron in slices prepared from mice 48 hours after deprivation. Example images show immunohistochemistry against GFP (left), GluA2 (middle) and GRIP1 (right). Scale bar: 2 μm. Bottom, fluorescence intensity traces measured in the numbered spines in each example of GFP (green), GluA2 (red), GRIP1 (magenta) and background (gray; 90 degree rotation of fluorescence image, not shown). Scale bars: 2 μm (horizontal) and 25 intensity units (vertical).

W,X) Spine intensity values of GluA2 (filled) and GRIP1 (open) for a subset of dendritic spines in inhibitory (W) and excitatory (X) neurons measured for the same spine in either the original immunohistochemical images or images rotated by 90 degrees. Each circle is the measure from an individual spine. (GluA2: Original versus rotated, W, $p<0.001$; X, $p<0.001$. GRIP1: Original versus rotated, W, $p<0.001$; X, $p<0.001$, Wilcoxon signed rank test).

Y) Percentage of imaged spines showing GluA2 (filled) and GRIP1 (open) intensity values that are greater than background (calculated from the 90 degree rotated immunohistochemistry image) for inhibitory (red) or excitatory (blue) cells in slices prepared from animals 48 hours after enucleation. Inhibitory, n=79 branches. Excitatory, n=62 branches.

Z) Spine GluA2 intensity normalized to background, then normalized to individual spine size for inhibitory (red) and excitatory (blue) neurons in slices prepared 48 hours after deprivation (red/blue) and from control mice (black). (Control versus deprived: Inhibitory, $p<0.001$; Excitatory, $p=0.001$, Mann Whitney Rank Sum Test). Inhibitory: deprived, n=998 spines; control, n=1887 spines. Excitatory: deprived, n=1025 spines; control, n=1450 spines.

Insets for summary data panels: mouse with objective is *in vivo* imaging experiment, slice with objective is *in vitro* imaging experiment, slice with electrode is *in vitro* electrophysiology experiment. For all panels, **$p<0.01$; ***$p<0.001$. Error bars, mean and s.e.m. For clarity, crossing axons have been removed from all images.

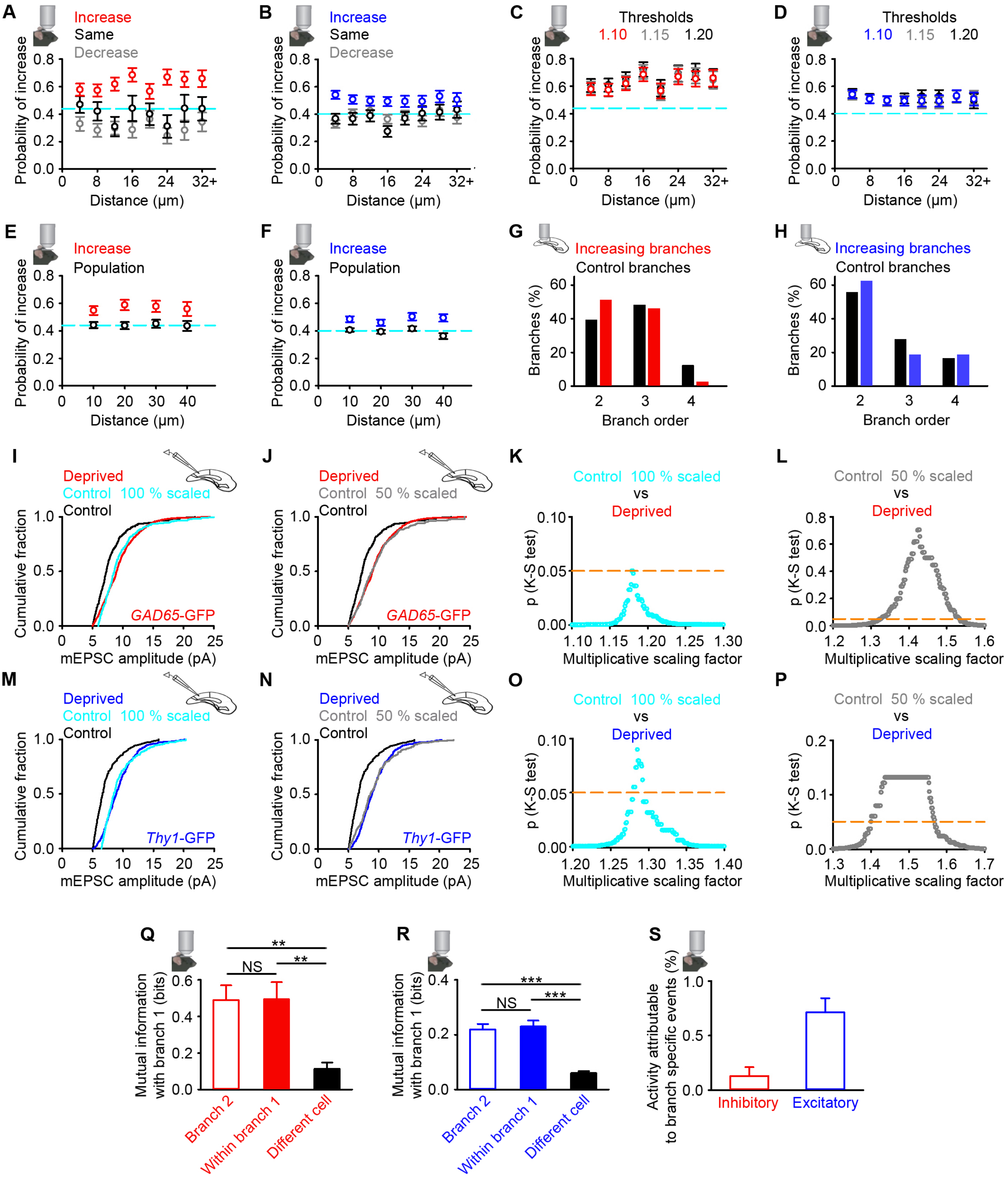

Supplementary Figure 2

Figure S2, Related to Figure 2.

A,B) Same cluster analysis as in Fig. 2B-C, but grouped by the behavior of the spine Sp_0 which either increases (blue/red, $Sp_0>1.1$), stays a similar size ('same', black, $1.1>Sp_0>0.9$) or decreases in size (gray, $Sp_0<0.9$). For each Sp_0 behavior, the fraction of spines at different distances from spine Sp_0 (4 µm bins) increasing for inhibitory (A, Interaction between behaviour and position, $p=0.829$; Behavior: Increase versus same, $p<0.001$; Increase versus decrease, $p<0.001$, Two-Way ANOVA with post-hoc test) and excitatory neurons (B, Interaction between behaviour and position, $p=0.726$; Behavior: Increase versus same, $p<0.001$; Increase versus decrease, $p<0.001$, Two-Way ANOVA with post-hoc test). Data are shown for 48 hours after enucleation normalized to baseline value for each spine. Cyan dashed line depicts proportion of all spines increasing. For panel A, n=31 branches. For panel B, n=24 branches.

C,D) For all Sp_0 spines exceeding an increased size threshold (1.1, 1.15, 1.2), the fraction of all neighbors a given distance (in 4 µm bins) away on the dendritic branch that also exceed the same size increase threshold as the Sp_0 spine (1.1, 1.15, 1.2) measured 48 hours post-enucleation and normalized to baseline for individual spines from branches in inhibitory (C, Threshold and distance, $p=0.487$, Two-Way ANOVA) and excitatory neurons (D, Threshold and distance, $p=0.845$, Two-Way ANOVA). Cyan dashed line depicts proportion of all spines increasing. For panel C, n=31 branches. For panel D, n=24 branches.

E,F) Same cluster analysis as in Fig. S2A-D and Fig. 2B-C for spines exceeding a threshold of 1.1 size increase within a given distance (in 10 µm bins) of a spine, Sp_0, 48 hours after enucleation normalized to baseline value. For Sp_0s that either exceed threshold 1.1 (red/blue) or the population average including all spines (black) for dendritic branches from inhibitory (E, Increasing versus population, $p<0.001$; 10 µm, $p=0.001$; 20 µm, $p<0.001$; 30 µm, $p=0.011$; 40 µm, $p=0.036$; Within increasing, 10 µm versus 40 µm, $p=0.854$; Two-Way ANOVA with post-hoc test) and excitatory neurons (F, Increasing versus population, $p<0.001$; 10 µm, $p=0.002$; 20 µm, $p=0.013$; 30 µm, $p=0.004$; 40 µm, $p<0.001$; Within increasing, 10 µm versus 40 µm, $p=0.767$; Two-Way ANOVA with post-hoc test). Cyan dashed line depicts proportion of all spines increasing. For panel E, n=31 branches. For panel F, n=24 branches.

G,H) Distribution of branch order (percentage of branches for each branch order out of all branches within a condition) for either inhibitory (G) or excitatory (H) neurons for branches showing an increase in spine size

(red/blue) and branches from sham enucleated control animals (black) from the same blindly collected and analyzed dataset. For panel G, control n=148 branches, deprived n=79 branches. For panel H, control n=62 branches, deprived n=62 branches.

I,J,M,N) Distribution of mEPSC amplitude for inhibitory neurons (I-J) and excitatory (M-N) neurons in slices prepared 48 hours after deprivation from control mice (black) with either 100% (I,M) or 50% (J,N) of mEPSC events from the control distribution multiplicatively scaled (cyan 100%, gray 50%) compared to the deprived distribution (red/blue). Data are shown for the best fit scaling factor (I, 1.179; J, 1.424; M, 1.285; N, 1.46). For panels I,J, control n=9 cells, deprived n=16 cells. For panels M,N, control n=10 cells, deprived n=15 cells.

K,L,O,P) Resulting p-values from Kolomogrov-Smirnov (K-S) tests (circles) for the multiplicative scaling factors used to scale either the entire (100%) control distribution (K,O) or 50% of the control distribution (L,P) and comparing it to the deprived distribution for either inhibitory (K-L) or excitatory neurons (O-P). Orange dashed line shows where p=0.05 (5% confidence interval). We tested the goodness of fit to the experimentally measured deprived distribution for the 50% scaled and for the 100% scaled control distributions using a Kullback-Leibler divergence statistic. We found a lower Kullback-Leibler value for the 50% scaled distribution, corresponding to less information lost and a better fit to the deprived distribution in both inhibitory (100% Scaled = 0.129 ± 0.003, 50% Scaled = 0.037 ± 0.001 Bits, p<0.001, t-test on Kullback-Leibler divergence scores) and excitatory (100% Scaled = 0.177 ± 0.001, 50% Scaled = 0.059 ± 0.001 Bits, p<0.001, t-test on Kullback-Leibler divergence scores) neurons.

Q,R) For GCaMP6f functional imaging measurements in behaving mice, the mutual information calculation with branch 1 for a dendritic branch sharing a branch point (Branch 2), a neighboring region 10 μm apart on the same dendritic branch (Within branch 1) or a dendrite in the same imaging region, but on a different cell (Different cell) in inhibitory neurons 24 hours post-enucleation (Q) or excitatory neurons 4 hours post-enucleation (R) (Q, red open (Branch 1-Branch 2) versus red filled (Branch 1-Within branch 1), p=0.970; red open versus black filled (Branch 1-Different cell), p=0.003; red filled versus black filled, p=0.005; R, blue open (Branch 1-Branch 2) versus blue filled (Branch 1-Within branch 1), p=0.686; blue open versus black filled (Branch 1-Different cell), p<0.001; blue filled versus black filled, p<0.001, One-Way ANOVA with post-hoc test). For panel Q, n=7 branch-1 cells, n=7 different cells. For panel R, n=6 branch-1 cells, n=6 different cells.

S) Activity attributable to branch specific events as a percentage of total overall activity in the branch for inhibitory (red) and excitatory (blue) branches in deprived animals. Inhibitory, n=7 branch pairs. Excitatory, n=6 branch pairs.

Insets for summary data panels: mouse with objective is *in vivo* imaging experiment, slice with objective is *in vitro* imaging experiment, slice with electrode is *in vitro* electrophysiology experiment. For all panels, NS=no significance; **p<0.01; ***p<0.001. Error bars, mean and s.e.m.

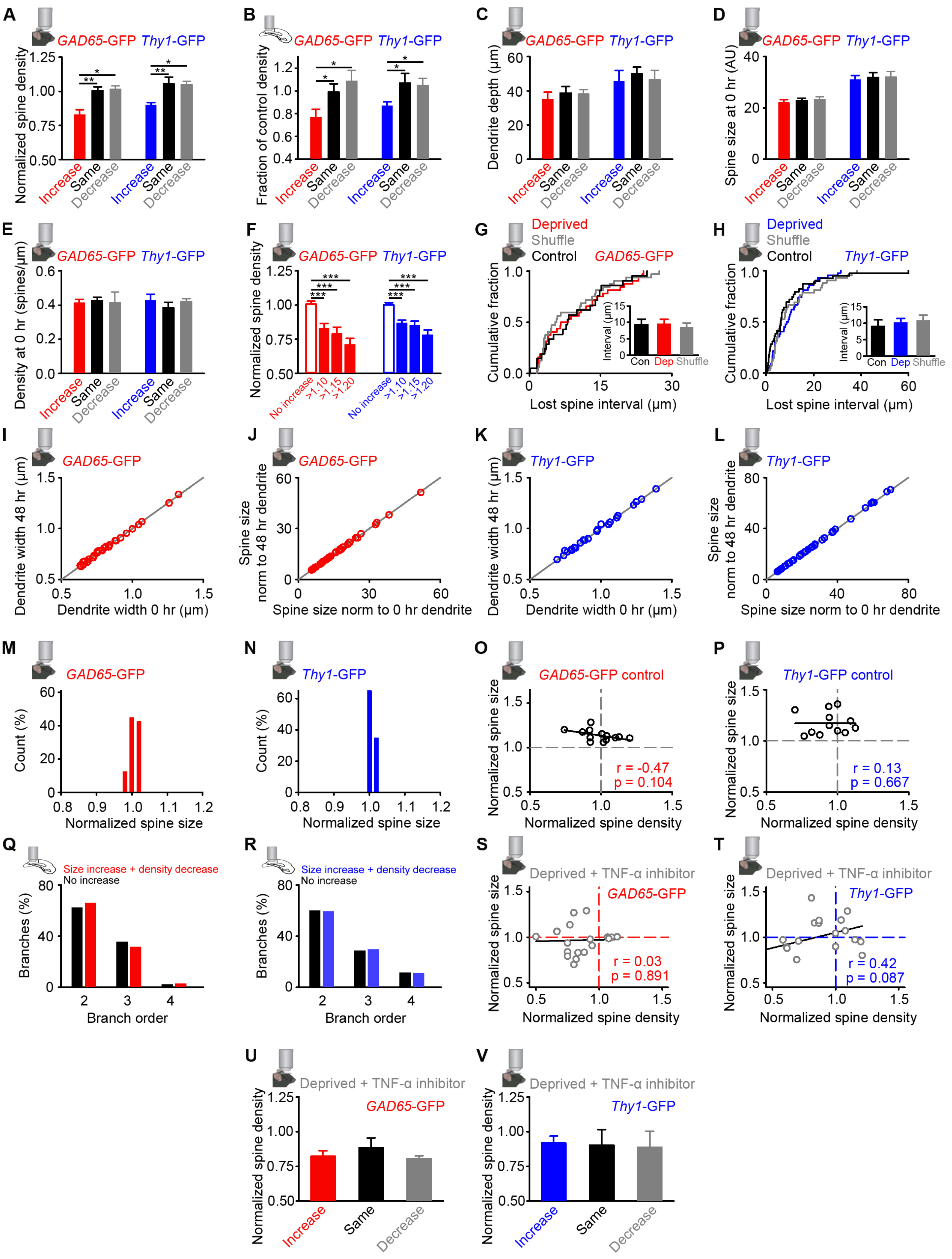

Supplementary Figure 3

Figure S3, Related to Figure 3.

A) Spine density 48 hours post-enucleation normalized to baseline for individual branches whose average post-enucleation spine size increases (red/blue, >1.1), stays a similar average size ('same', black, between 0.9 and 1.1) or decreases in size (gray, <0.9) relative to their individual baseline in inhibitory (Increase versus same, p=0.002; Increase versus decrease, p=0.021, One-Way ANOVA with post-hoc test) and excitatory neurons (Increase versus same, p=0.005; Increase versus decrease, p=0.028, One-Way ANOVA with post-hoc test). Inhibitory, n=31 branches; Excitatory, n=24 branches.

B) Fraction of control spine density (fraction of average control value) for individual branches whose spines increase in size (red/blue, >1.1), stay a similar average size ('same', black, between 0.9 and 1.1) or decrease in size (gray, <0.9) relative to control values (see Methods) measured from slices prepared from mice 48 hours after enucleation in inhibitory (Increase versus same, p=0.047; Increase versus decrease, p=0.010, One-Way ANOVA with post-hoc test) and excitatory neurons (Increase versus same, p=0.026; Increase versus decrease, p=0.049, One-Way ANOVA with post-hoc test). Inhibitory, n=79 branches; Excitatory, n=62 branches.

C) Cortical depth (distance from the surface of the brain to branch midpoint) of dendritic branches whose average post-enucleation spine size increases (red/blue, >1.1), stays a similar average size ('same', black, between 0.9 and 1.1) or decreases in size (gray, <0.9) relative to their individual baseline after enucleation in inhibitory (red, p=0.812, One-Way ANOVA) and excitatory (blue, p=0.846, One-Way ANOVA) neurons in deprived animals measured with *in vivo* imaging. Inhibitory, n=31 branches. Excitatory, n=24 branches.

D) Average stable spine size prior to deprivation for dendritic branches whose average post-enucleation spine size increases (red/blue, >1.1), stays a similar average size ('same', black, between 0.9 and 1.1) or decreases in size (gray, <0.9) relative to their individual baseline in inhibitory (red, p=0.427, One-Way ANOVA on ranks) and excitatory (blue, p=0.373, One-Way ANOVA on ranks) neurons measured with *in vivo* imaging. Note that this measure is for stable spines that will not be lost following deprivation. Inhibitory, n=31 branches. Excitatory, n=24 branches.

E) Spine density prior to deprivation for dendritic branches whose average post-enucleation spine size increases (red/blue, >1.1), stays a similar average size ('same', black, between 0.9 and 1.1) or decreases in size (gray, <0.9)

relative to their individual baseline in inhibitory (red, p=0.927, One-Way ANOVA) and excitatory (blue, p=0.659, One-Way ANOVA) neurons measured with *in vivo* imaging. Inhibitory, n=31 branches. Excitatory, n=24 branches.

F) Spine density at 48 hours post-enucleation normalized to baseline within dendrite for branches that do not show average spine size increases (<1.1, individual spines 48 hours post-enucleation normalized to baseline, then averaged across the dendritic branch) and those that show an average spine size change of greater than 1.1, 1.15 or 1.2 for inhibitory (red) and excitatory (blue) neurons. (Inhibitory: No increase versus >1.1, p<0.001; No increase versus >1.15, p<0.001; No increase versus >1.2, p<0.001. Excitatory: No increase versus >1.1, p<0.001; No increase versus >1.15, p<0.001; No increase versus >1.2, p<0.001, One-Way ANOVA with post-hoc test). Inhibitory, n=31 branches. Excitatory, n=52 branches (includes data from Fig. 1E and Fig. 3E).

G,H) Cumulative distribution of interspine intervals for lost spines across all time points in inhibitory (G) and excitatory (H) neurons in deprived (red/blue) and control (black) animals and for deprived branches that underwent a spatial shuffle (gray). Insets, mean spine loss interspine interval. (Inhibitory: p=0.815; Excitatory: p=0.314, One-Way ANOVA on Ranks). For panel G, n=31 branches. For panel H, n=24 branches.

I,K,) Dendrite width measured prior to (0 hours) and 48 hours after enucleation for inhibitory (I) and excitatory (K) neurons. Each circle is a single dendrite. (0 hours versus 48 hours: I, p=0.197; K, p=0.984, paired t-test). For panel I, n=31 branches. For panel K, n=24 branches.

J,L) Spine size measured at 0 hours normalized to the adjacent dendrite taken from the image at either 0 hours or 48 hours after enucleation. Each circle is a single spine. (0 hours versus 48 hours: J, p=0.477; L, p=0.421, paired t-test). For panel J, n=40 spines. For panel L, n=40 spines.

M,N) Distribution of 0 hour spine size measured using the dendrite at 48 hours normalized to the same 0 hour spine size measured using the dendrite at 0 hours in inhibitory (M, 1.000 ± 0.002 normalized spine size) and excitatory neurons (N, 1.000 ± 0.001 normalized spine size). For panel M, n=40 spines. For panel N, n=40 spines.

O,P) Spine density versus average spine size normalized to baseline for dendritic branches in inhibitory (O) and excitatory (P) neurons that undergo an average increase in spine size (but not necessarily "increasing branches", see Methods) in sham-enucleated animals measured with chronic *in vivo* imaging. Spine density and size are measured 48 hours (O) or 8 hours (P) after sham-enucleation and normalized to baseline for individual branches (density) and individual spines (size). Normalized spine size is then averaged across the branch. (O, r=-0.47, p=0.104; P, r=0.13, p=0.667, Pearson's correlation). For panel O, n=13 branches. For panel P, n=12 branches.

Q,R) Distribution of branch order (percentage of branches for each branch order out of all branches within a condition) for either inhibitory (Q) or excitatory (R) neurons measured from slices prepared from mice 48 hours after enucleation. Branches showing an increase in spine size and a decrease in spine density (red/blue) and branches that do not show an increase (black) from the same blindly collected and analyzed dataset. For panel Q, n=79 branches. For panel R, n=62 branches.

S,T) For deprived animals that also received injections of the TNF-α inhibitor, normalized spine density versus average normalized stable spine size measured using repeated *in vivo* imaging. Measured 48 hours after enucleation and normalized to baseline for individual dendritic branches (density) or individual spines (size). Normalized spine size was then averaged for each branch. Dendrites in inhibitory (S) and excitatory (T) neurons (S, r=0.03, p=0.891; T, r=0.42, p=0.087, Pearson's correlation). For panel S, n=19 branches. For panel T, n=18 branches.

U,V) Spine density in deprived animals that were injected with the TNF-α inhibitor 48 hours post-enucleation normalized to baseline for individual branches whose average spine size increases (red/blue, >1.1), stays a similar average size ('same', black, between 0.9 and 1.1) or decreases in size (gray, <0.9) in inhibitory (U, Increase versus same, p=0.658; Increase versus decrease, p=0.676, One-Way ANOVA with post-hoc test) and excitatory (V, Increase versus same, p=0.895; Increase versus decrease, 0.809, One-Way ANOVA with post-hoc test) neurons. For panel U, n=19 branches. For panel V, n=18 branches.

Insets for summary data panels: mouse with objective is *in vivo* imaging experiment, slice with objective is *in vitro* imaging experiment. For all panels, *p<0.05; **p<0.01; ***p<0.001. Error bars, mean and s.e.m.

Statistical Comparisons for Figure 1			Inhibitory		Excitatory	
**Panel**	**Comparison**	**Test**	**p value**	**n value**	**p value**	**n value**
1D,E	Time and experimental condition, overall interaction	Two-Way ANOVA with post-hoc test on log-transformed data	p = 0.015	control n=40 branches<br>deprived n=31 branches<br>deprived+TNF-α inhibitor n=19 branches	p = 0.006	control n=34 branches<br>deprived n=24 branches<br>deprived+TNF-α inhibitor n=18 branches
	Control vs deprived at 0 hrs		p = 0.359		p = 0.417	
	Control vs deprived + TNF-α inhibitor at 0 hrs		p = 0.312		p = 0.588	
	Deprived vs deprived + TNF-α inhibitor at 0 hrs		p = 0.670		p = 0.291	
	Control vs deprived at 24 hrs		p = 0.560		p < 0.001	
	Control vs deprived + TNF-α inhibitor at 24 hrs		p = 0.708		p = 0.627	
	Deprived vs deprived + TNF-α inhibitor at 24 hrs		p = 0.512		p = 0.008	
	Control vs deprived at 48 hrs		p = 0.008		p < 0.001	
	Control vs deprived + TNF-α inhibitor at 48 hrs		p = 0.064		p = 0.923	
	Deprived vs deprived + TNF-α inhibitor at 48 hrs		p < 0.001		p < 0.001	
1D,E	0 vs 24 hrs deprived	One-Way Repeated Measures ANOVA with post-hoc test on log-transformed data	p = 0.638	control n=40 branches<br>deprived n=31 branches<br>deprived+TNF-α inhibitor n=19 branches	p = 0.045	control n=34 branches<br>deprived n=24 branches<br>deprived+TNF-α inhibitor n=18 branches
	0 vs 24 hrs deprived+TNF-α inhibitor		p = 0.655		p = 0.959	
	0 vs 24 hrs control		p = 0.224		p = 0.264	
	0 vs 48 hrs deprived		p = 0.021		p = 0.003	
	0 vs 48 hrs deprived+TNF-α inhibitor		p = 0.204		p = 0.624	
	0 vs 48 hrs control		p = 0.092		p = 0.061	
1G	Control vs deprived	One-Way ANOVA with post-hoc test	p = 0.015	control n=9 cells<br>deprived n=16 cells<br>deprived+TNF-α inhibitor n=10 cells		
	Deprived vs deprived + TNF-α inhibitor		p < 0.001			
	Control vs deprived + TNF-α inhibitor		p = 0.237			
1H	Control vs deprived	One-Way ANOVA on Ranks with post-hoc test			p < 0.001	control n=10 cells<br>deprived n=15 cells<br>deprived+TNF-α inhibitor n=13 cells
	Deprived vs deprived + TNF-α inhibitor				p = 0.031	
	Control vs deprived + TNF-α inhibitor				p = 0.325	
1J	Control vs deprived	t-test	p = 0.029	control n=148 branches deprived n=79 branches	p = 0.031	control n=62 branches deprived n=62 branches
1K	Control vs deprived	t-test	p = 0.021	control n=148 branches deprived n=79 branches	p = 0.043	control n=62 branches deprived n=62 branches

Table S1. Statistical comparisons for Figure 1, Related to Figure 1.

Statistical Comparisons for Figure 2			Inhibitory		Excitatory	
**Panel**	**Comparison**	**Test**	**p value**	**n value**	**p value**	**n value**
**2B,C**	Increasing vs population over all distance	Two-Way ANOVA with post-hoc test	p < 0.001	n = 31 branches	p < 0.001	n = 24 branches
	Increasing vs population at 4 µm		p = 0.039		p < 0.001	
	Increasing vs population at 8 µm		p = 0.018		p = 0.010	
	Increasing vs population at 12 µm		p = 0.002		p = 0.012	
	Increasing vs population at 16 µm		p = 0.002		p = 0.002	
	Increasing vs population at 20 µm		p = 0.035		p = 0.005	
	Increasing vs population at 24 µm		p < 0.001		p = 0.008	
	Increasing vs population at 28 µm		p = 0.049		p = 0.026	
	Increasing vs population at 32+ µm		p = 0.012		p = 0.029	
	Increasing vs population at different branch		p = 0.558		p = 0.438	
	Within increasing: 4 µm vs 28 µm		p = 0.996		p = 0.998	
	Increasing vs shuffle at 4 µm		p = 0.875		p = 0.141	
	Increasing vs shuffle at 8 µm		p = 0.635		p = 0.709	
	Increasing vs shuffle at 12 µm		p = 0.552		p = 0.994	
	Increasing vs shuffle at 16 µm		p = 0.207		p = 0.682	
	Increasing vs shuffle at 20 µm		p = 0.666		p = 0.887	
	Increasing vs shuffle at 24 µm		p = 0.996		p = 0.509	
	Increasing vs shuffle at 28 µm		p = 0.635		p = 0.397	
	Increasing vs shuffle at 32+ µm		p = 0.688		P = 0.414	
**2D**	One vs both	One-Way ANOVA with post-hoc test	p < 0.001	n = 94 branch pairs	p = 0.019	n = 138 branch pairs
	One vs neither		p = 0.002		p = 0.003	
**2E**	One vs both	One-Way ANOVA with post-hoc test	p = 0.029	n = 100 branch pairs	p = 0.031	n = 72 branch pairs
	One vs neither		p = 0.027		p = 0.022	
**2H,I**	Branch 1 and branch 2	Spearman's correlation	r = 0.90 p < 0.001	example	r = 0.91 p < 0.001	example
	Branch 1 and different cell		r = -0.16 p < 0.001		r = 0.19 p < 0.001	
**2J,K**	Branch 2 vs within branch 1 vs different cell	One-Way repeated measures ANOVA	p = 0.264	n = 7 branch 1 cells n = 7 different cells	p = 0.145	n = 6 branch 1 cells n = 6 different cells
**2L,M**	Branch 2 vs within branch 1	One-Way ANOVA with post-hoc test	p = 0.746	n = 7 branch 1 cells n = 7 different cells	p = 0.900	n = 6 branch 1 cells n = 6 different cells
	Within branch 1 vs different cell		p < 0.001		p < 0.001	
	Branch 2 vs different cell		p < 0.001		p < 0.001	
**2O**	Branch specific vs all events	t-test	p < 0.001	n = 7 branch pairs n = 166 total calcium events	p < 0.001	n = 6 branch pairs n=140 total calcium events

**2P**	Branch specific vs all events	t-test	$p < 0.001$	n=7 branch pairs n=166 total calcium events	$p < 0.001$	n=6 branch pairs n=140 total calcium events
**2Q**	Deprived vs control	t-test	$p = 0.982$	deprived n = 7 branch pairs control n = 7 branch pairs	$p = 0.945$	deprived n = 6 branch pairs control n = 7 branch pairs

Table S2. Statistical comparisons for Figure 2, Related to Figure 2. For panel 2D, there is a higher n for branch pair comparisons because some cells have more than two branches and for excitatory cells, we have included data from Fig. 1E and Fig. 3E.

Statistical Comparisons for Figure 3			Inhibitory		Excitatory	
**Panel**	**Comparison**	**Test**	**p value**	**n value**	**p value**	**n value**
**3C**	Increase vs no increase	t-test	p < 0.001	n = 31 branches	p = 0.004	n = 24 branches
**3D**	Density 24 hrs	Repeated measures ANOVA with post-hoc test	p < 0.001	n = 31 branches		
	Density 48 hrs		p < 0.001			
	Size 24 hrs	Repeated measures ANOVA with post-hoc test on log-transformed data	p = 0.638			
	Size 48 hrs		p = 0.021			
**3E**	Density 4 hrs	Repeated measures ANOVA with post-hoc test			p < 0.001	n = 28 branches
	Density 8 hrs				p < 0.001	
	Size 4 hrs	Repeated measures ANOVA with post-hoc test on log-transformed data			p = 0.570	
	Size 8 hrs				p = 0.002	
**3F,G**	Normalized spine size vs normalized spine density	Pearson's correlation	r = -0.65<br>p < 0.001	n = 31 branches	r = -0.65<br>p < 0.001	n = 28 branches
**3I**	Increase vs no increase	t-test	p = 0.023	n = 79 branches	p = 0.043	n = 62 branches
**3J,K**	Fraction of control size vs fraction of control density	Pearson's correlation	r = -0.33<br>p < 0.001	n = 79 branches	r = -0.30<br>p = 0.032	n = 62 branches
**3L,M**	Density 24 hrs	Repeated measures ANOVA with post-hoc test	p = 0.003	n = 19 branches	p = 0.359	n = 18 branches
	Density 48 hrs		p < 0.001		p = 0.027	
**3N**	Increase vs no increase	t-test	p = 0.771	n = 19 branches	p = 0.832	n = 18 branches

Table S3. Statistical comparisons for Figure 3, Related to Figure 3.
